# Supplementary material for: Cardiorespiratory response to early rehabilitation in critically ill adults: A secondary analysis of a randomised controlled trial
Source: PLoS One. 2022 Feb 3;17(2):e0262779. doi: 10.1371/journal.pone.0262779 (PMC8812982; doi:10.1371/journal.pone.0262779)
Supplement: S1 File — (PDF) [file pone.0262779.s001.pdf]

Supplemental file 1

Rehabilitation details

	Intervention group				Control group
	**Movement exercise**	**Resistance training**	**Endurance training (cycling)**	**Mobilisation**	
**Type**	Passive or assistive range of movement with tactile facilitation to excite movement	Three standardized exercises for upper and lower limbs: elbow flexion, arm push, external shoulder rotation, foot dorsiflexion, bridging, leg raise	Motor-assisted bed-cycle ergometer	In-bed mobilisation (positioning, sitting), out-of-bed mobilisation (edge of bed, chair, standing, walking)	European standard Physiotherapy including early mobilisation, respiratory therapy and passive or active exercises.
**Time**	**STEP 1:** after study inclusion	Less than 25% support during assistive movement exercises	**STEP 2:** started if STEP 1 was tolerated	**STEP 3:** started if STEP 2 tolerated and no medical contraindications	Medical referral (daily screening on weekdays by physiotherapist)
**Frequency**	Once daily on weekdays	Upper limbs on Monday, Wednesday, Friday. Lower limbs on Tuesday and Thursday.	Once daily on weekdays	At least once daily on weekdays	Once daily on weekdays
**Intensity**	3-10 repetitions, 1-2 set, each joint, all directions	8-12 repetitions, 2-5 sets with 2min rest, 50-70% of estimated one-repetition maximum (weights starting from 450g or manual resistance)	If passive: maximum of 20min, 20 cycles/min<br>If active: maximum of 60min, level 6, target BORG level 11-13	Continued progressively to achieve maximal possible mobility level	individually tailored
**Who**	Certified physiotherapist	Certified physiotherapist	Certified physiotherapist (training was supervised)	Certified physiotherapist	Certified physiotherapist
**Tailoring**	If partial movement possible, assist to full range of motion	Individual exercises prescribed by responsible therapist	Achieve active participation before adjusting assistance [a], increase time before resistance [b]	Progressed mobility level depending on tolerance and stability	Determined by physiotherapist

[a] After 20min of active-assistive cycling decrease assistance (each session) to level 2

[b] Increase time to 30min, then increase resistance every second day to level 6, then increase time

Intervention code

Each physiotherapy session was allocated an intervention code with 7 positions that expressed the interventions performed during that session. The 7 positions were allocated as follows (intervention examples are given in brackets):

1. **Resistance training** (muscle strength training against resistance)
2. **Cycling** (in-bed cycling)
3. **Movement exercise** (passive, assisted or active range-of-motion exercises)
4. **Mobilisation** (sitting up in-bed or edge-of-bed, transferring to a chair, standing or walking)
5. **Respiratory therapy** (deep breathing exercises, cough augmentation, airway clearance)
6. **Dysphagia therapy** (tracheostomy management (i.e., cuff deflation), swallowing training)
7. **Others** (relaxation, massage, lymph drainage)

Thus, a physiotherapy session that included resistance training and mobilisation was coded as 1001000 or a session with cycling and respiratory therapy as 0100100.

Treatment categories (intervention type)

Subsequently, the intervention code was allocated to one of the seven predefined treatment categories, here referred to as ‘intervention type’, that in our unit resemble common treatment packages in the critically ill. The following intervention codes were allocated to each of these seven groups:

1. **Group: “cycling” (in-bed cycling plus optional: resistance training, movement exercise, respiratory management, others)**
   0100000
   0100011
   0100101
   0100111
   0110000
   0110001
   0110101
   1100000
   1110000
   1110101

2. **Group: “mobilisation” (mobilisation plus optional: resistance training, movement exercise, respiratory management, others)**
   0001000
   0001001
   0001011
   0001101
   0001111
   1011000

3. **Group: “respiratory management” (respiratory therapy, dysphagia therapy or relaxation)**
   0000011
   0000101
   0000111
   0000001

The treatment modality (active, passive, mixed patient participation) of these interventions was not recorded by the treating physiotherapist. To reduce missing data, this was therefore coded as “passive” for the category “respiratory management”.

4. **Group: "exercise" (movement exercise plus optional: resistance training, others)**
   0010000
   0010001
   1010000
   1010001

5. **Group "exercise and respiratory management" (combination of group 3 and 4)**
   0010101
   1010011
   1010101
   1010111

6. **Group "complex exercise and mobilisation" (combination of group 2, 3 and 4)**
   0011000
   0011001
   1001011
   0011101
   1001101
   1001111
   1011001
   1011011
   1011101
   1011111

7. **Group "complex cycling and mobilisation" (combination of group 1, 2, 3 and 4)**
   0101000
   0101101
   0111000
   0111101
   1101000
   1111101

Mixed-effects statistical models

$$y_{ijt} = \alpha_0 + \alpha_i + \gamma_0 y_{ij0} + \gamma_{cv}\mathrm{CV}_{y,ij0} + \beta^T X_{ij} + \varepsilon_{ijt},$$

where y_{ijt} is the physiological value of interest for subject i during or after ($t = 1$ or 2 respectively) the physiotherapy session j, y_{ij0} is the measurement of interest before the session, $\mathrm{CV}_{y,ij0}$ is the CV of the measurement of interest before the session, X_{ij} is the vector of covariates for subject i at session j, α_0 is the intercept to estimate, β is the parameters vector of fixed effects to estimate, $\alpha_i \sim N(0, \sigma_\alpha^2)$ is the random effect handling the correlation among the same subject' measurements across multiple sessions with σ_α^2 to estimate and $\varepsilon_{ijt} \sim N(0, \sigma_\varepsilon^2)$ is the residuals term independent and normally distributed with variance parameter σ_ε^2 to estimate. Parameters γ_0 and γ_{cv} are also estimated, however, according to Vickers (2001), were not the main focus and present only to adjust for pre-session physiological values in order to make statistical inferences about the covariates with the most impact on the physiological changes from before to during or from before to after physiotherapy.

Fixed effects, confidence intervals and p-values computed in R with packages "lmer" and "lmerTest". We excluded physiological values from HR and MAP with a corresponding zero CV to account for cardiac pacing.
