# Supplementary material for: Cardiorespiratory response to early rehabilitation in critically ill adults: A secondary analysis of a randomised controlled trial
Source: PLoS One. 2022 Feb 3;17(2):e0262779. doi: 10.1371/journal.pone.0262779 (PMC8812982; doi:10.1371/journal.pone.0262779)
Supplement: S2 File — (PDF) [file pone.0262779.s002.pdf]

## Supplemental file 2

**S1 Table.** Physiological values (median and coefficient of variability) for before, during and after physiotherapy including summary of differences from before to during and before to after physiotherapy.

| Variable | n | median [25%, 75%] | mean (95% CI) |
|---|---|---|---|
| median MAP before | 626 | 71 [63, 82] | 72 (46, 99) |
| median MAP during | 620 | 72 [64, 83] | 74 (48, 100) |
| median MAP after | 618 | 71.5 [63, 81] | 72 (46, 99) |
| median HR before | 664 | 92 [81, 104] | 92 (59, 125) |
| median HR during | 662 | 93 [83, 104] | 94 (61, 127) |
| median HR after | 659 | 92.5 [82, 104] | 93 (60, 126) |
| median $SpO_2$ before | 663 | 96 [95, 98] | 96 (92, 101) |
| median $SpO_2$ during | 661 | 96 [95, 98] | 96 (91, 101) |
| median $SpO_2$ after | 658 | 96 [95, 98] | 96 (91, 101) |
| median $VO_2$ before | 345 | 297 [246, 354] | 309 (128, 489) |
| median $VO_2$ during | 342 | 304 [258, 368] | 320 (138, 503) |
| median $VO_2$ after | 336 | 299 [252, 348] | 308 (149, 466) |
| median MV before | 516 | 10 [9, 13] | 11 (5, 17) |
| median MV during | 515 | 11 [9, 14] | 12 (5, 18) |
| median MV after | 510 | 10 [9, 13] | 11 (5, 17) |
| MAP CV before | 626 | 21 [14, 32] | 29 (-26, 84) |
| MAP CV during | 620 | 20 [12, 31] | 25 (-13, 63) |
| MAP CV after | 618 | 14 [9, 23] | 19 (-14, 51) |
| HR CV before | 664 | 8 [4, 13] | 11 (-9, 30) |
| HR CV during | 662 | 7 [4, 13] | 10 (-10, 30) |
| HR CV after | 659 | 6 [3, 10] | 8 (-8, 24) |
| $SpO_2$ CV before | 663 | 3 [2, 5] | 4 (-3, 10) |
| $SpO_2$ CV during | 661 | 3 [2, 5] | 4 (-4, 11) |
| $SpO_2$ CV after | 658 | 2 [1, 3] | 2 (-3, 8) |
| $VO_2$ CV before | 345 | 28 [18, 53] | 41 (-27, 108) |
| $VO_2$ CV during | 342 | 24 [15, 43] | 36 (-30, 102) |
| $VO_2$ CV after | 336 | 17 [11, 30] | 27 (-28, 82) |
| MV CV before | 516 | 32 [21, 47] | 36 (-7, 79) |
| MV CV during | 515 | 29 [18, 45] | 34 (-9, 76) |
| MV CV after | 510 | 22 [14, 37] | 27 (-8, 62) |
| difference MAP: during - before | 620 | 2 [-2, 5] | 2 (-10, 13) |
| difference MAP: after - before | 618 | -1 [-4, 4] | 0 (-14, 14) |
| difference HR: during - before | 662 | 1 [-1, 4] | 2 (-8, 11) |
| difference HR: after - before | 659 | 1 [-2, 3] | 1 (-10, 12) |
| difference SpO2 during - before | 660 | 0 [-1, 1] | 0 (-3, 3) |

| | | | |
|---|---|---|---|
| difference SpO2: after - before | 657 | 0 [-1, 1] | 0 (-3, 3) |
| difference VO2 during - before | 339 | 9 [-2, 25] | 12 (-65, 89) |
| difference VO2: after - before | 333 | 2 [-11, 18] | 2 (-79, 83) |
| difference MV: during - before | 508 | 0 [0, 1] | 1 (-2, 4) |
| difference MV: after - before | 503 | 0 [-1, 1] | 0 (-3, 3) |
| difference CV MAP; during - before | 620 | -2 [-12, 8] | -4 (-66, 58) |
| difference CV MAP: after - before | 618 | -7 [-16, 2] | -10 (-68, 48) |
| difference CV HR: during - before | 662 | -1 [-4, 3] | -1 (-20, 19) |
| difference CV HR: after - before | 659 | -1 [-5, 1] | -2 (-20, 16) |
| difference CV SpO2: during - before | 660 | 0 [-1, 1] | 0 (-9, 9) |
| difference CV SpO2: after - before | 657 | -1 [-3, 0] | -1 (-9, 6) |
| difference CV VO2: during - before | 339 | -4 [-22, 10] | -6 (-91, 80) |
| difference CV VO2: after - before | 333 | -8 [-28, 2] | -14 (-95, 67) |
| difference CV MV: during - before | 508 | -2 [-13, 9] | -2 (-46, 41) |
| difference CV MV: after - before | 503 | -8 [-20, 3] | -9 (-53, 35) |

**Measurement units.** MAP (mmHg), HR (bpm), VO2 (ml/min), SpO2 (%), MV (l/min), CV (%).

**S2 Figure.** Correlations of before, during and after physiotherapy medians (md) and coefficient of variability (CV) for the following physiological values: heart rate (HR), mean arterial pressure (MAP), oxygen consumption ($VO_2$), peripheral oxygen saturation ($SpO_2$) and minute ventilation (MV).

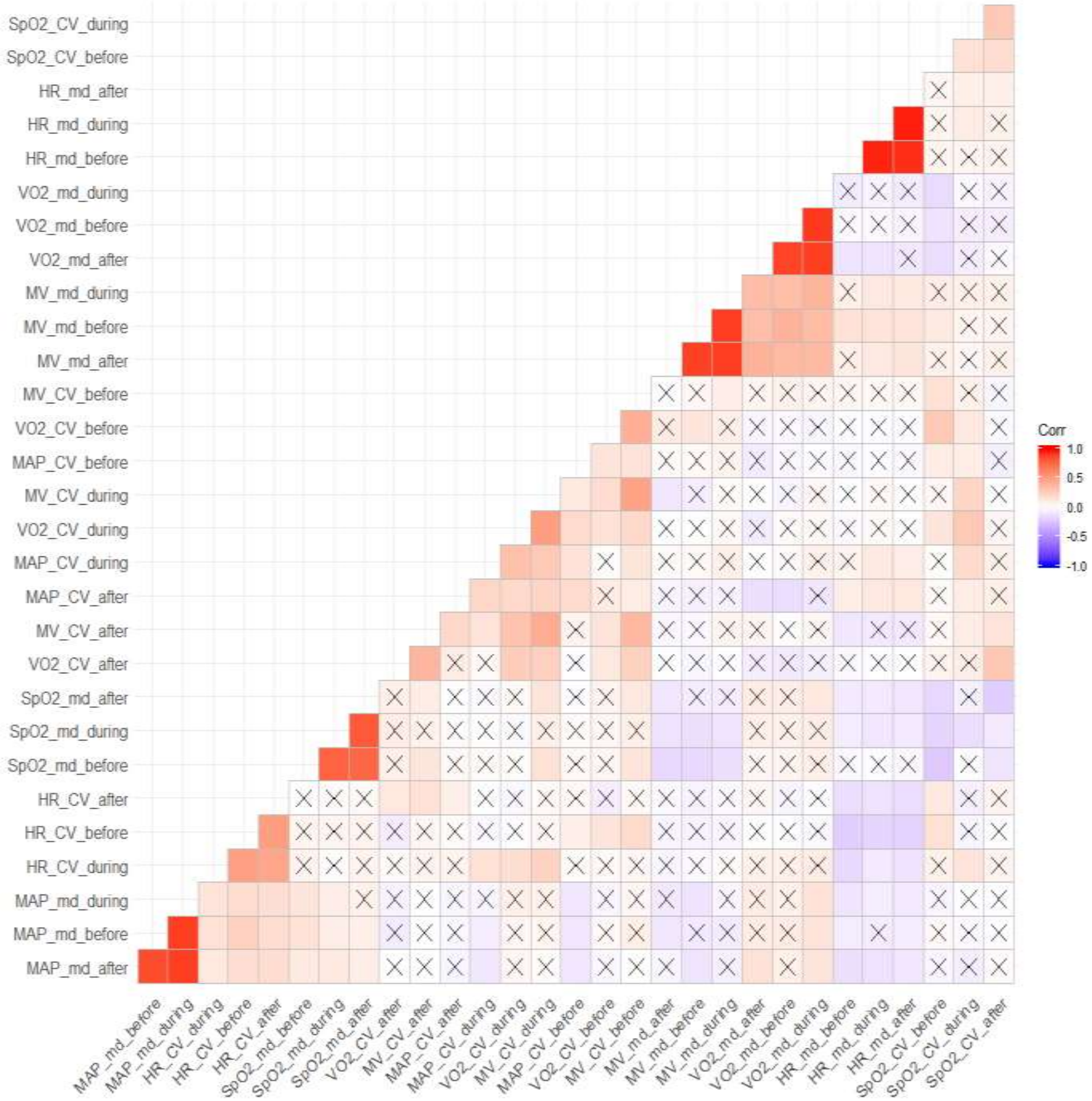

**S3 Figure.** Differences between coefficient of variability (CV) of physiological values from before to during/after physiotherapy.

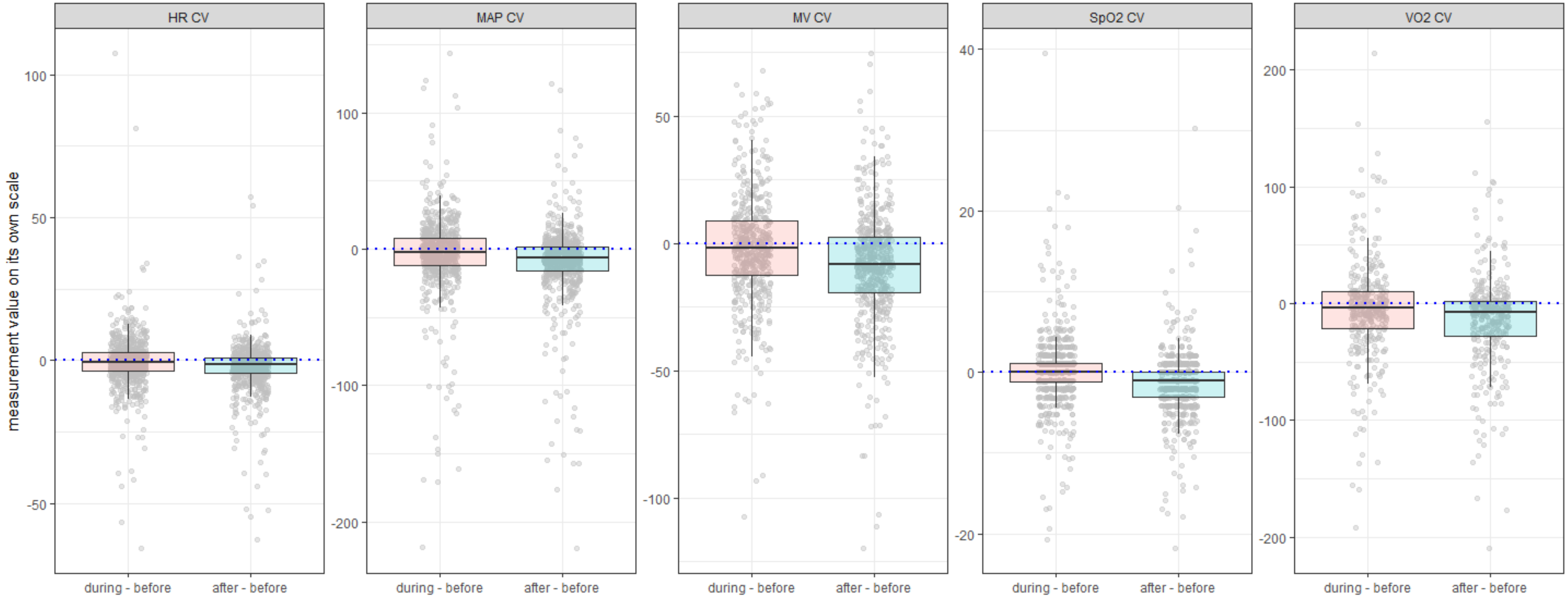


**Legend.** CV-differences were calculated as “during minus before” (training) and “after minus before” (recovery) physiotherapy for all physiological values. Measurement units: HR (bpm), MAP (mmHg), MV (l/min), SpO2 (%), VO2 (ml/min).

**S4 Figure.** Trajectories of CVs of physiological values according to session type.

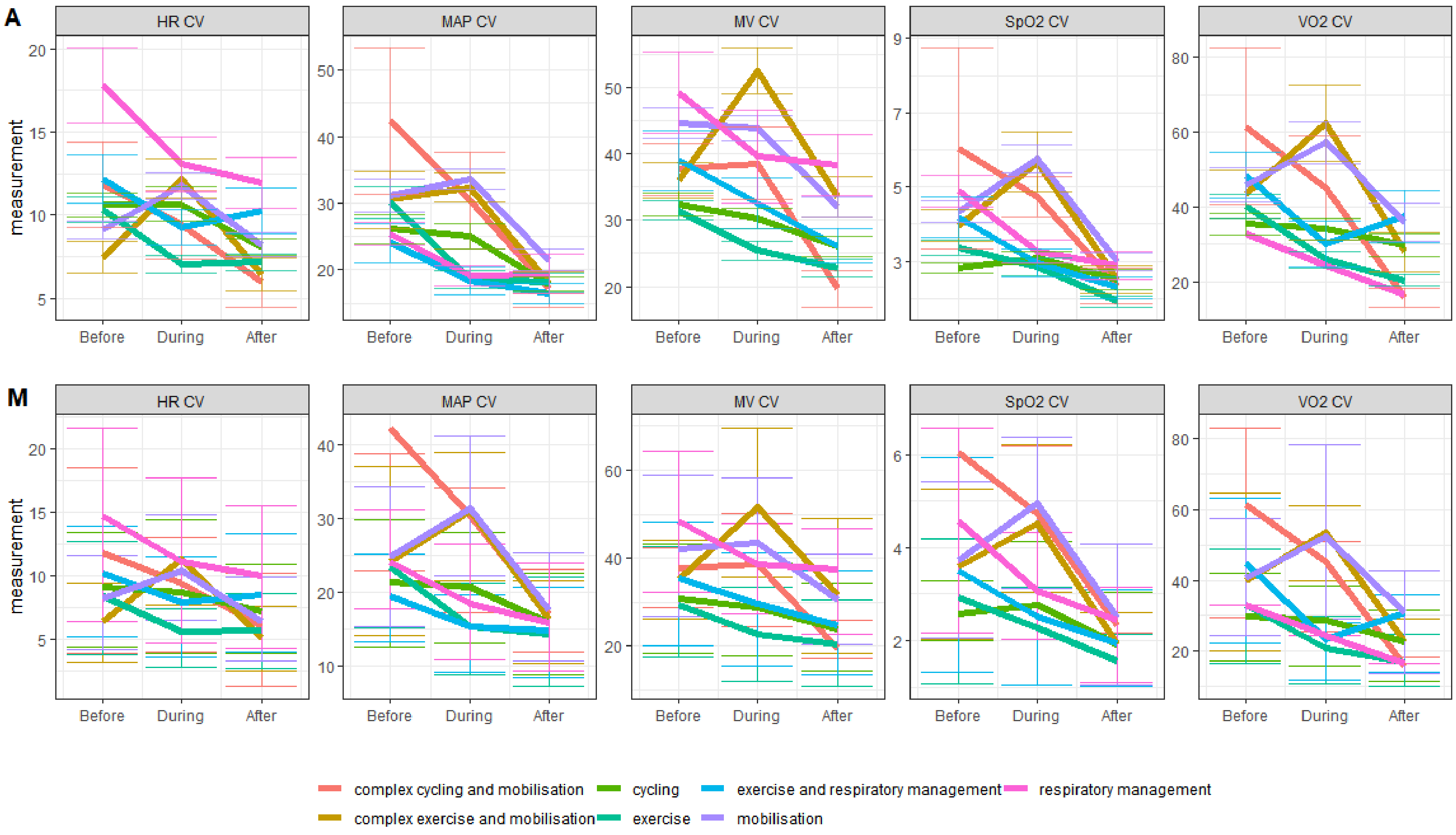


**Legend.** A) Average trajectories with standard errors. M) median trajectories with IQR of physiological measurements. Standard errors are computed under the wrong assumption of independences among all the observations. Measurement units: HR (bpm), MAP (mmHg), MV (l/min), $SpO_2$ (%), $VO_2$ (ml/min).

**S2 Table.** Overview of clinically relevant changes (>10%) of physiological variables.

| Variable | Clinically relevant changes (>10%) [n (%)] | Sensitivity analysis for cut-off >20%, [n (%)] |
|---|---|---|
| **$VO_2$ 'training' (N=339)** | | |
| Not clinically relevant | 251 (74.0%) | 311 (91.7%) |
| Clinically relevant | 88 (26.0%) | 28 (8.3%) |
| **VO2 'recovery' (N=333)** | | |
| Not clinically relevant | 246 (73.9%) | 305 (91.6%) |
| Clinically relevant | 87 (26.1%) | 28 (8.4%) |
| **MV 'training' (N=508)** | | |
| Not clinically relevant | 327 (64.4%) | 443 (87.2%) |
| Clinically relevant | 181 (35.6%) | 65 (12.8%) |
| **MV 'recovery' (N=503)** | | |
| Not clinically relevant | 333 (66.2%) | 447 (88.9%) |
| Clinically relevant | 170 (33.8%) | 56 (11.1%) |
| **MAP 'training' (N=620)** | | |
| Not clinically relevant | 485 (78.2%) | 598 (96.5%) |
| Clinically relevant | 135 (21.8%) | 22 (3.5%) |
| **MAP 'recovery' (N=618)** | | |
| Not clinically relevant | 444 (71.8%) | 582 (94.2%) |
| Clinically relevant | 174 (28.2%) | 36 (5.8%) |
| **HR 'training' (N=662)** | | |
| Not clinically relevant | 619 (93.5%) | 655 (98.9%) |
| Clinically relevant | 43 (6.5%) | 7 (1.1%) |
| **HR 'recovery' (N=659)** | | |
| Not clinically relevant | 605 (91.8%) | 648 (98.3%) |
| Clinically relevant | 54 (8.2%) | 11 (1.7%) |
| **SpO2 'training' (N=660)** | | |
| Not clinically relevant | 659 (99.8%) | 659 (99.8%) |
| Clinically relevant | 1 (0.2%) | 1 (0.1%) |
| **SpO2 'recovery' (N=657)** | | |
| Not clinically relevant | 660 (100%) | 657 (100%) |
| Clinically relevant | 0 (0%) | 0 (0%) |

**Legend.** Denominator N corresponds to available sessions.

**S5 Figure.** Correlations between explanatory variables.

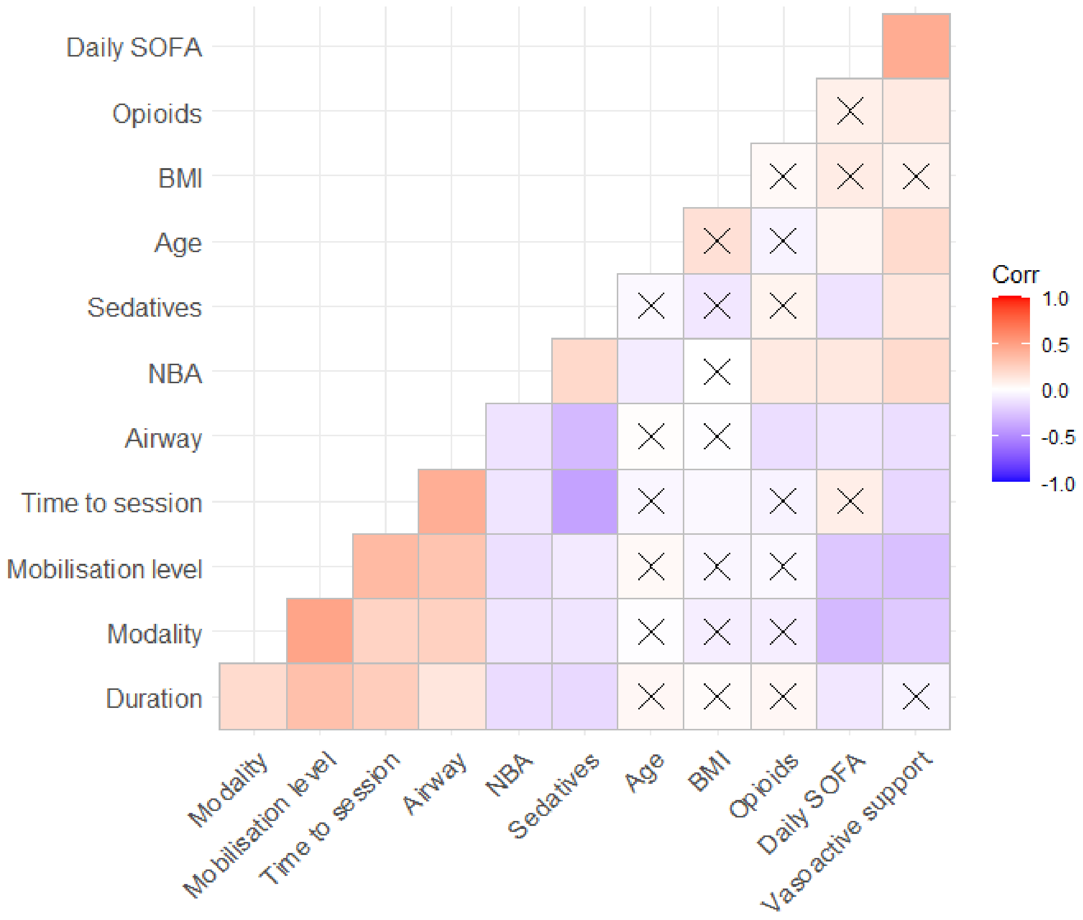


**Legend.** All correlations were below 0.5, "X" indicates a non-significant correlation. **Abbreviations.** SOFA = Sequential Organ Failure Assessment; BMI = Body Mass Index ($kg/m^2$); NBA = Neuromuscular Blocking Agents.

**S3 Table.** Co-occurrence of 'mobilisation level' and 'session type'.

| | in-bed | edge-of-bed | out-of-bed |
|---|---|---|---|
| respiratory management | 66 | 0 | 0 |
| cycling | 160 | 0 | 0 |
| exercise | 193 | 0 | 0 |
| exercise and respiratory management | 54 | 0 | 0 |
| complex cycling and mobilisation | 8 | 1 | 1 |
| complex exercise and mobilisation | 3 | 37 | 15 |
| mobilisation | 4 | 112 | 62 |

**S4 Table.** Estimated fixed effect of explanatory variables on SpO2 (%) during and after physiotherapy.

| **Explanatory variables** | | **$SpO_2$ during (95%-CI)** | **$SpO_2$ after (95%-CI)** |
|---|---|---|---|
| Number of sessions fitted in the model | | 570 | 568 |
| Age (years) [a] | | 0.001 (-0.01, 0.01) | 0.004 (-0.01, 0.02) |
| Gender (male is reference) | | -0.08 (-0.31, 0.13) | -0.15 (-0.43, 0.12) |
| Body Mass Index ($kg/m^2$) [a] | | 0.01 (-0.02, 0.03) | 0.01 (-0.02, 0.03) |
| Daily SOFA score (0-24) [a] | | -0.01 (-0.04, 0.02) | -0.01 (-0.04, 0.03) |
| Session duration (min) [a] | | -0.01 (-0.02, 0.001) | 0.01 (-0.01, 0.02) |
| Time from ICU admission to start of session (days) [a] | | 0.01 (-0.04, 0.02) | 0.01 (-0.001, 0.02) |
| Session type (exercise is reference) | | | |
| | | 0.297 [c] | 0.273 [c] |
| | cycling | 0.12 (-0.18, 0.42) | -0.07 (-0.45, 0.30) |
| | mobilisation | 0.22 (-0.86, 1.31) | -0.82 (-2.15, 0.55) |
| | respiratory management | 0.05 (-0.41, 0.50) | -0.43 (-1.00, 0.14) |
| | exercise and respiratory management | **0.52 (0.13, 0.92)** | 0.35 (-0.15, 0.84) |
| | complex cycling and mobilisation | 0.21 (-0.66, 1.09) | -0.30 (-1.39, 0.79) |
| | complex exercise and mobilisation | 0.33 (-0.73, 1.37) | -0.97 (-2.28, 0.35) |
| Treatment modality (passive is reference) | | | |
| | | 0.554 [c] | 0.888 [c] |
| | mixed | 0.30 (-0.25, 0.86) | 0.13 (-0.55, 0.82) |
| | active | 0.01 (-0.26, 0.29) | -0.05 (-0.39, 0.31) |
| Mobilisation level (in-bed is reference) | | | |
| | | **<0.001 [c]** | 0.330 [c] |
| | edge-of-bed | -0.64 (-1.72, 0.43) | 0.56 (-0.80, 1.88) |
| | out-of-bed | -1.03 (-2.15, 0.09) | 0.13 (-1.28, 1.51) |
| Airway support (none is reference) | | | |
| | | **<0.001 [c]** | **0.021 [c]** |
| | tracheostomy | 0.39 (0.01, 0.78) | 0.05 (-0.43, 0.53) |
| | endotracheal tube | 0.05 (-0.31, 0.40) | -0.44 (-0.88, -0.00) |
| Opiates during session (none is reference) | | -0.09 (-0.47, 0.32) | -0.13 (-0.62, 0.37) |
| Vasoactive support during session (none is reference) | | -0.14 (-0.39, 0.11) | -0.18 (-0.49, 0.13) |
| Sedatives during session (none is reference) | | 0.08 (-0.20, 0.37) | -0.07 (-0.42, 0.29) |
| Neuromuscular blocking agents during session (none is reference) | | 0.14 (-0.16, 0.43) | -0.07 (-0.45, 0.29) |

[a] per one-unit increase (for continuous variables)

[b] defined as maximum minus minimum divided by mean multiplied by 100

[c] likelihood test for overall significance of categorical variables

**Legend.** Effects of explanatory variables need to be considered under the assumption of 'all other covariates being constant'.
