# Supplementary material for: Cardiorespiratory response to early rehabilitation in critically ill adults: A secondary analysis of a randomised controlled trial
Source: PLoS One. 2022 Feb 3;17(2):e0262779. doi: 10.1371/journal.pone.0262779 (PMC8812982; doi:10.1371/journal.pone.0262779)
Supplement: S3 File — (DOCX) [file pone.0262779.s003.docx]

**Supplemental file 3**

1. **Adverse events and therapy discontinuation**

The four adverse events included two transient oxygen desaturations and two temporary unstable haemodynamics. Physiotherapists most frequently discontinued a session (n=23) due to exceedance of individually set limits (7 [30%]), lack of patient cooperation (4 [17%]) fatigue (3 [13%]), pain (2 [9%]), unplanned procedure/intervention (2 [9%]) and other causes (5 [22%]: 2x difficulties with settings of in-bed cycle, 1x calorimetry not recorded, 1x dizziness, 1x difficulties with settings of continuous renal replacement therapy). Most of these events (n=27) were related to mobilisation (13 [48%]), the rest occurred during cycling (7 [26%]) or exercise and respiratory management (7 [26%]). Patients in these sessions were all females with a median age of 60 years [25%, 75% quantile: 51, 68] and a SOFA score of 8 [5, 14] on that day. Session duration was a median of 18min [15, 25]. During the occurrence 13 [48%] were intubated, 5 [19%] tracheotomised, and 9 [33%] breathed spontaneously, while 25 [93%] received opiates, 26 [96%] sedation, 15 [56%] vasoactive support, and 6 [22%] neuromuscular blocking agents.

1. **Investigation of the two outliers in Fig 1**

The first patient experienced a substantial drop in SpO_2_ from before (99%) to during (76%) physiotherapy, but recovered after the session (96%). On that day, this 56-year-old female had received opioids and sedatives. She had a SOFA score of 11 with an oxygenation-index of 270mmHg and a Glasgow Coma Scale of 9. The session continued for 15min with an edge-of-bed mobilisation and respiratory management. This session has not been reported as an adverse event nor was the therapy discontinued, though the patient had two other therapy discontinuations.

The second patient experienced a decline in VO_2_ from before (703ml/min) to after (290ml/min) physiotherapy. The participant was a 71-year-old female with a SOFA score of 9 (oxygenation-index 168mmHg, Glasgow Coma Scale 14) receiving opioids and sedatives on that day. Physiotherapy duration was 45min and included an active out-of-bed mobilisation without any reported adverse events or therapy discontinuations. We found a large within-subject variation for the original 2-median values (S5 Table).

**S5 Table.** Example of within-subject variation of VO_2_ (ml/min) in one subject over physiotherapy duration.

| before | during | after |
| --- | --- | --- |
| 918.30 |  |  |
| 416.80 |  |  |
| 723.70 |  |  |
| 900.10 |  |  |
| 888.30 |  |  |
| 711.60 |  |  |
| 402.00 |  |  |
| 861.80 |  |  |
| 706.60 |  |  |
| 545.80 |  |  |
| 469.20 |  |  |
| 656.20 |  |  |
| 698.40 |  |  |
| 750.00 |  |  |
| 593.20 |  |  |
| 587.30 | 587.30 |  |
|  | 740.10 |  |
|  | 787.70 |  |
|  | 934.70 |  |
|  | 677.70 |  |
|  | 336.20 |  |
|  | 470.70 |  |
|  | 366.10 |  |
|  | 986.30 |  |
|  | 519.20 |  |
|  | 664.70 |  |
|  | 786.40 |  |
|  | 509.10 |  |
|  | 553.00 |  |
|  | 345.40 |  |
|  | 318.90 |  |
|  | 330.60 |  |
|  | 523.80 |  |
|  | 805.50 |  |
|  | 421.50 |  |
|  |  | 270.60 |
|  |  | 268.10 |
|  |  | 621.40 |
|  |  | 309.30 |

1. **Factors for clinically relevant variations**

We used a multilevel logistic regression model with the following predictors to investigate factors related to clinically relevant variations:

- **Mobilisation level:** out-/edge-of-bed versus **in-bed=reference**
- **Treatment modality:** mixed/active versus **passive=reference**
- **Session duration** (one-unit increase)
- **Daily SOFA score** (one-unit increase)
- Adapted **session type**: **group1**=cycling, **group2**= mobilisation (including: ‘complex cycling and mobilisation’; ‘complex exercise and mobilisation’, **group3=exercise=reference** (including ‘respiratory management’; ‘exercise and respiratory management’)
- Age (one-unit increase)

We did not investigate SpO_2_ because there were only a few clinically relevant changes. Similarly, there were insufficient events to investigate all the above factors for HR. We therefore excluded the predictor ‘session type’ in this model. Results are reported in the tables below (S6 to S13 table).

**S6 Table.** Factors for clinically relevant variations in VO_2_ during rehabilitation (n=94, sessions=312).

|  | OR | Lower 95% CI | Upper 95% CI | p-value |
| --- | --- | --- | --- | --- |
| Mobilisation level | 2.29 | 0.39 | 13.44 | 0.36 |
| Treatment modality | 1.47 | 0.78 | 2.77 | 0.24 |
| Session duration | 1.02 | 0.99 | 1.05 | 0.22 |
| Daily SOFA score | 1.03 | 0.97 | 1.09 | 0.36 |
| Session type ‘group 2’ ^a^ | 0.91 | 0.17 | 4.81 | 0.91 |
| Session type ‘group 1’ ^a^ | 0.70 | 0.13 | 3.75 | 0.68 |
| Age | 1.00 | 0.98 | 1.02 | 0.87 |

^a^ Overall ANOVA test for the categorical variable ‘session type’: p=0.73.

**S7 Table.** Factors for clinically relevant variations in VO_2_ after rehabilitation (n=94, sessions=308).

|  | OR | Lower 95% CI | Upper 95% CI | p-value |
| --- | --- | --- | --- | --- |
| Mobilisation level | 4.74 | 0.46 | 48.42 | 0.19 |
| Treatment modality | 1.19 | 0.61 | 2.31 | 0.61 |
| Session duration | 1.04 | 1.00 | 1.08 | 0.05 |
| Daily SOFA score | 0.99 | 0.92 | 1.06 | 0.75 |
| Session type ‘group 2’ ^a^ | 2.92 | 0.31 | 27.10 | 0.35 |
| Session type ‘group 1’ ^a^ | 2.66 | 0.30 | 23.86 | 0.38 |
| Age | 1.01 | 0.99 | 1.03 | 0.44 |

^a^ Overall ANOVA test for the categorical variable ‘session type’: p=0.58.

**S8 Table.** Factors for clinically relevant variations in MV during rehabilitation (n=103, sessions=442).

|  | OR | Lower 95% CI | Upper 95% CI | p-value |
| --- | --- | --- | --- | --- |
| Mobilisation level | 1.10 | 0.28 | 4.40 | 0.89 |
| Treatment modality | 1.62 | 0.97 | 2.72 | 0.06 |
| Session duration | 1.00 | 0.98 | 1.03 | 0.79 |
| Daily SOFA score | 0.99 | 0.94 | 1.05 | 0.82 |
| Session type ‘group 2’ ^a^ | 0.47 | 0.13 | 1.75 | 0.26 |
| Session type ‘group 1’ ^a^ | 0.51 | 0.14 | 1.93 | 0.32 |
| Age | 1.00 | 0.98 | 1.02 | 0.76 |

^a^ Overall ANOVA test for the categorical variable ‘session type’: p=0.54.

**S9 Table.** Factors for clinically relevant variations in MV after rehabilitation (n=103, sessions=437).

|  | OR | Lower 95% CI | Upper 95% CI | p-value |
| --- | --- | --- | --- | --- |
| Mobilisation level | 2.30 | 0.44 | 11.88 | 0.32 |
| Treatment modality | 0.88 | 0.53 | 1.46 | 0.62 |
| Session duration | 1.03 | 1.00 | 1.05 | 0.02 |
| Daily SOFA score | 0.93 | 0.89 | 0.98 | <0.001 |
| Session type ‘group 2’ ^a^ | 2.35 | 0.48 | 11.49 | 0.29 |
| Session type ‘group 1’ ^a^ | 2.62 | 0.53 | 12.95 | 0.24 |
| Age | 1.01 | 1.00 | 10.3 | 0.12 |

^a^ Overall ANOVA test for the categorical variable ‘session type’: p=0.43.

**S10 Table.** Factors for clinically relevant variations in MAP during rehabilitation (n=107, sessions=535).

|  | OR | Lower 95% CI | Upper 95% CI | p-value |
| --- | --- | --- | --- | --- |
| Mobilisation level | 0.85 | 0.19 | 3.81 | 0.84 |
| Treatment modality | 1.15 | 0.68 | 1.93 | 0.61 |
| Session duration | 1.00 | 0.98 | 1.03 | 0.70 |
| Daily SOFA score | 1.02 | 0.96 | 1.08 | 0.47 |
| Session type ‘group 2’ ^a^ | 0.64 | 0.15 | 2.72 | 0.54 |
| Session type ‘group 1’ ^a^ | 0.57 | 0.13 | 2.50 | 0.46 |
| Age | 1.02 | 1.00 | 1.04 | 0.06 |

^a^ Overall ANOVA test for the categorical variable ‘session type’: p=0.76.

**S11 Table.** Factors for clinically relevant variations in MAP after rehabilitation (n=107, sessions=534).

|  | OR | Lower 95% CI | Upper 95% CI | p-value |
| --- | --- | --- | --- | --- |
| Mobilisation level | 2.59 | 0.50 | 13.34 | 0.26 |
| Treatment modality | 1.15 | 0.71 | 1.84 | 0.57 |
| Session duration | 1.00 | 0.98 | 1.03 | 0.65 |
| Daily SOFA score | 1.00 | 0.95 | 1.05 | 0.98 |
| Session type ‘group 2’ ^a^ | 1.56 | 0.31 | 7.80 | 0.59 |
| Session type ‘group 1’ ^a^ | 1.58 | 0.31 | 8.00 | 0.58 |
| Age | 1.00 | 0.98 | 1.02 | 0.92 |

^a^ Overall ANOVA test for the categorical variable ‘session type’: p=0.85.

**S12 Table.** Factors for clinically relevant variations in HR during rehabilitation (n=107, sessions=571) without the predictor ‘session type’.

|  | OR | Lower 95% CI | Upper 95% CI | p-value |
| --- | --- | --- | --- | --- |
| Mobilisation level | 2.84 | 1.01 | 7.93 | 0.05 |
| Treatment modality | 2.01 | 0.80 | 5.04 | 0.14 |
| Session duration | 1.00 | 0.96 | 1.03 | 0.82 |
| Daily SOFA score | 0.93 | 0.82 | 1.05 | 0.25 |
| Age | 0.98 | 0.95 | 1.02 | 0.41 |

**S13 Table.** Factors for clinically relevant variations in HR after rehabilitation (n=107, sessions=569) without the predictor ‘session type’.

|  | OR | Lower 95% CI | Upper 95% CI | p-value |
| --- | --- | --- | --- | --- |
| Mobilisation level | 0.66 | 0.26 | 1.70 | 0.39 |
| Treatment modality | 2.06 | 1.00 | 4.23 | 0.05 |
| Session duration | 1.01 | 0.98 | 1.04 | 0.53 |
| Daily SOFA score | 0.90 | 0.82 | 0.99 | 0.04 |
| Age | 1.03 | 1.00 | 1.06 | 0.03 |
