# Supplementary material for: Cardiorespiratory response to early rehabilitation in critically ill adults: A secondary analysis of a randomised controlled trial
Source: PLoS One. 2022 Feb 3;17(2):e0262779. doi: 10.1371/journal.pone.0262779 (PMC8812982; doi:10.1371/journal.pone.0262779)
Supplement: S6 File — (PDF) [file pone.0262779.s006.pdf]

Analysis code and regression output to Eggmann et al.: Cardiorespiratory response to early rehabilitation in critically ill adults: a secondary analysis of a randomised controlled trial

Date: 2021-08-20

Background

The following report reproduces the main tables and figures (Table 1-3, Figure 1 and 2) of the manuscript from *Eggmann et al.: Cardiorespiratory response to early rehabilitation in critically ill adults: a secondary analysis of a randomised controlled trial.*

The underlying data (*data.csv*) is provided within the submission. Note that continuous variables have been rounded to 2 decimal places, such that estimates might negligibly differ from the original numbers reported in the manuscript.

We used the statistical software R:

```
##                _
## platform       x86_64-apple-darwin17.0
## arch           x86_64
## os             darwin17.0
## system         x86_64, darwin17.0
## status
## major          4
## minor          0.2
## year           2020
## month          06
## day            22
## svn rev        78730
## language       R
## version.string R version 4.0.2 (2020-06-22)
## nickname       Taking Off Again
```

for producing this report.

Used packages and reading data

```
library(tidyverse)
library(kableExtra)
library(haven)
library(lme4)
library(lmerTest)
library(reporttools)
library(cowplot)

data <- read_csv(paste0(data_path, "data.csv"))
```

Table 1

```r
tableNominal(data.frame(data %>% select(sex, rand_group, phtp_type, Rx_modal, Mob_level,
                                        Rx_abb, Rx_AE, Airway, relax, vaso, opia, sed)),
             cap = "Categorical variables.", cumsum = F)
```

Variable	Levels	n	%
sex	female	36	33.3
	male	72	66.7
	all	108	100.0
rand_group	control	53	49.1
	intervention	55	50.9
	all	108	100.0
phtp_type	complex cycling and mobilisation	10	1.4
	complex exercise and mobilisation	55	7.7
	cycling	160	22.4
	exercise	193	27.0
	exercise and respiratory management	54	7.5
	mobilisation	178	24.9
	respiratory management	66	9.2
	all	716	100.0
Rx_modal	active	187	30.7
	mixed	22	3.6
	passive	401	65.7
	all	610	100.0
Mob_level	edge-of-bed	150	20.9
	in-bed	488	68.2
	out-of-bed	78	10.9
	all	716	100.0
Rx_abb	No	693	96.8
	Yes	23	3.2
	all	716	100.0
Rx_AE	No	712	99.4
	Yes	4	0.6
	all	716	100.0
Airway	none	118	16.5
	tracheostomy	271	37.9
	tube	327	45.7
	all	716	100.0
relax	no	615	86.3
	yes	98	13.7
	all	713	100.0
vaso	no	342	48.0
	yes	371	52.0
	all	713	100.0
opia	no	51	7.2
	yes	662	92.8
	all	713	100.0
sed	no	174	24.4
	yes	539	75.6
	all	713	100.0

Table 1: Categorical variables.

```r
tableContinuous(data.frame(data %>% select(age, bmi, apache_2, sofa_score, days_incl,
                                           LOS_ICU, sessions_n, in_study_tm_first,
                                           in_study_tm, Rx_duration, Rx_sofa)),
                stats=c("n", "median", "q1", "q3", "mean", "s"),
                cap = "Continuous variables.")
```

Variable	n	$\tilde{x}$	q_1	q_3	$\bar{x}$	s
age	108	66.7	55.1	74.4	63.0	15.2
bmi	108	26.4	23.7	29.6	27.2	5.0

apache_2	108	22.0	17.8	27.2	22.8	7.2
sofa_score	108	8.0	6.0	11.0	8.9	3.8
days_incl	108	1.8	0.9	2.6	1.9	1.2
LOS_ICU	108	7.0	4.6	13.9	11.2	11.3
sessions_n	108	3.0	2.0	8.0	6.6	9.1
in_study_tm_first	108	2.0	1.4	3.1	2.5	1.8
in_study_tm	716	8.6	3.9	19.6	15.0	20.3
Rx_duration	716	22.0	16.8	30.0	24.2	11.7
Rx_sofa	713	8.0	5.0	12.0	8.8	4.4

Table 2: Continuous variables.

```r
fig_data <-
  bind_rows(data %>% select(dMAP_d, dMAP_a, phtp_type, record_id) %>% pivot_longer(cols=1:2),
            data %>% select(dHR_d, dHR_a, phtp_type, record_id) %>% pivot_longer(cols=1:2),
            data %>% select(dSpO2_d, dSpO2_a, phtp_type, record_id) %>% pivot_longer(cols=1:2),
            data %>% select(dVO2_d, dVO2_a, phtp_type, record_id) %>% pivot_longer(cols=1:2),
            data %>% select(dMV_d, dMV_a, phtp_type, record_id) %>% pivot_longer(cols=1:2),
            )

fig_data$time <- "during - before"
fig_data$time[grepl("_a", fig_data$name)] <- "after - before"

fig_data$time <- factor(fig_data$time, levels=c("during - before", "after - before"))

fig_data$name <- as.character(fig_data$name)
fig_data$name[which(fig_data$name %in% c("dMAP_d", "dMAP_a"))] <- "MAP median"
fig_data$name[which(fig_data$name %in% c("dHR_d", "dHR_a"))] <- "HR median"
fig_data$name[which(fig_data$name %in% c("dSpO2_d", "dSpO2_a"))] <- "SpO2 median"
fig_data$name[which(fig_data$name %in% c("dVO2_d", "dVO2_a"))] <- "VO2 median"
fig_data$name[which(fig_data$name %in% c("dMV_d", "dMV_a"))] <- "MV median"

boxplot_fig <- ggplot(fig_data, aes(x = time, y = value)) +
  geom_jitter(width = 0.15, alpha = 0.4, col = "grey") +
  geom_boxplot(outlier.shape = NA,  alpha = 0.2) +
  facet_wrap(. ~ name, scales='free', ncol = 6) + theme_bw() +
  ylab("measurement value on its own scale") + xlab("") +
  geom_hline(yintercept=0, linetype="dotted", color = "blue", size=1) +
  theme(legend.position = "none", axis.text.x = element_text(angle=45, hjust=1, vjust=1))

boxplot_fig
```

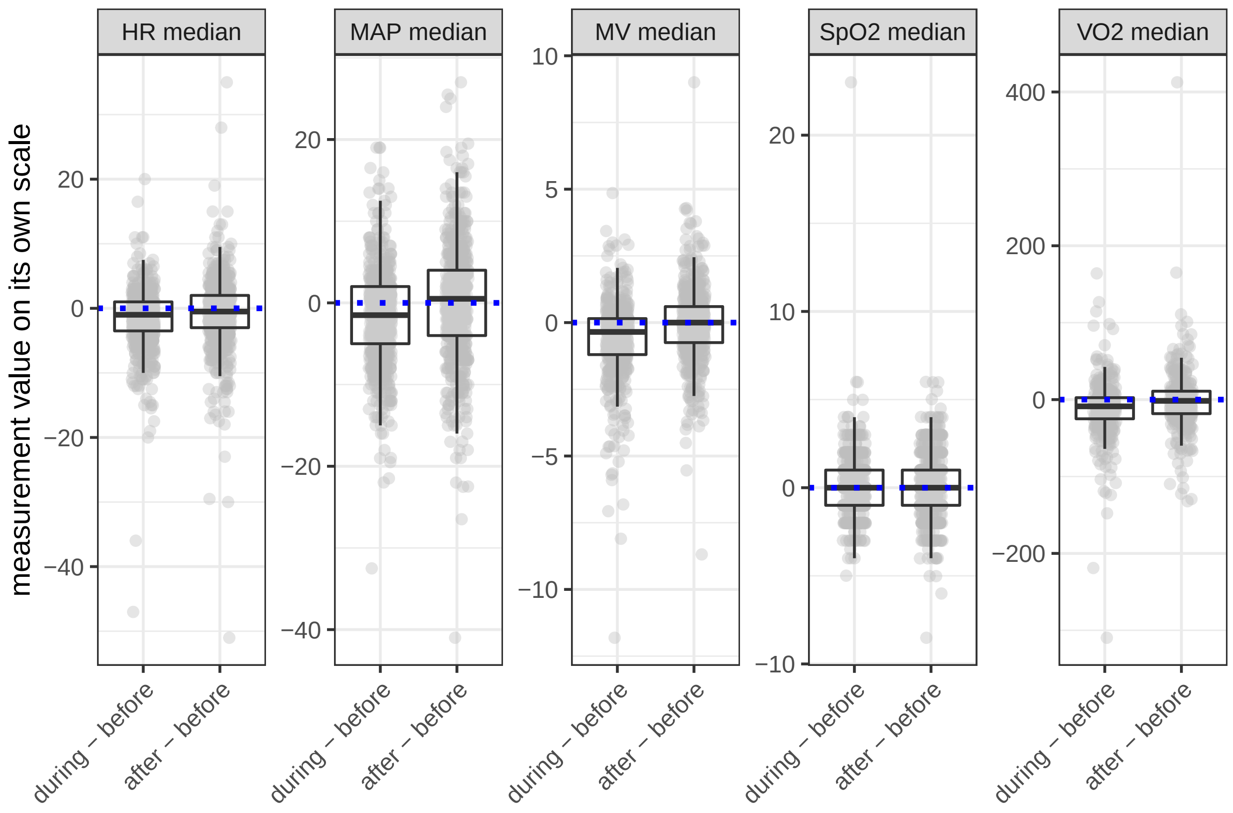

Figure 2

```
fig_data <-
  bind_rows(
    data %>% select(VO2_md_during, VO2_md_after, VO2_md_prior, phtp_type, record_id)
    %>% pivot_longer(cols=1:3),
    data %>% select(MV_md_during, MV_md_after, MV_md_prior, phtp_type, record_id)
    %>% pivot_longer(cols=1:3),
    data %>% select(MAP_md_during, MAP_md_after, MAP_md_prior, phtp_type, record_id)
    %>% pivot_longer(cols=1:3),
    data %>% select(HR_md_during, HR_md_after, HR_md_prior, phtp_type, record_id)
    %>% pivot_longer(cols=1:3),
    data %>% select(SpO2_md_during, SpO2_md_after, SpO2_md_prior, phtp_type, record_id)
    %>% pivot_longer(cols=1:3),
  )

fig_data$time <- 1
fig_data$time[grepl("_during", fig_data$name)] <- 2
fig_data$time[grepl("_after", fig_data$name)] <- 3

fig_data$name <- as.character(fig_data$name)
fig_data$name[which(fig_data$name %in%
                      c("MAP_md_prior", "MAP_md_during", "MAP_md_after"))] <- "MAP median"
fig_data$name[which(fig_data$name %in%
                      c("HR_md_prior", "HR_md_during", "HR_md_after"))] <- "HR median"
```

```
fig_data$name[which(fig_data$name %in%
                      c("SpO2_md_prior", "SpO2_md_during", "SpO2_md_after"))] <- "SpO2 median"
fig_data$name[which(fig_data$name %in%
                      c("VO2_md_prior", "VO2_md_during", "VO2_md_after"))] <- "VO2 median"
fig_data$name[which(fig_data$name %in%
                      c("MV_md_prior", "MV_md_during", "MV_md_after"))] <- "MV median"

fig_data$phtp_type <- as.factor(as.character(fig_data$phtp_type))

med_mean_plots <- ggplot(data = fig_data, aes(x = time, y = value, group = phtp_type,
                           linetype=phtp_type)) + geom_line(stat = "summary", fun = mean) +
  scale_x_continuous(name="", breaks = c(1,2,3), labels=c("Before", "During", "After")) +
  ylab("measurement") + facet_wrap(.~ name, scales='free', ncol = 5) +  theme_bw() +
  theme(legend.position="none") + labs(colour= " ")+scale_linetype("")

med_med_plots <-
  ggplot(data = fig_data, aes(x = time, y = value, group = phtp_type, linetype=phtp_type)) +
  geom_line(stat = "summary", fun = function(z) {mean(z, trim = 0.1)})+  theme_bw() +
  scale_x_continuous(name="", breaks = c(1,2,3),  labels=c("Before", "During", "After")) +
  ylab("measurement") + facet_wrap(.~ name, scales='free', ncol = 5) +
  theme(legend.position="bottom") + labs(colour= " ")+scale_linetype("")

fig_comb <-  plot_grid(med_mean_plots, med_med_plots, ncol = 1, labels = c("A", "M"),
                     rel_heights = c(0.8, 1), label_x = c(0,0))

fig_comb
```

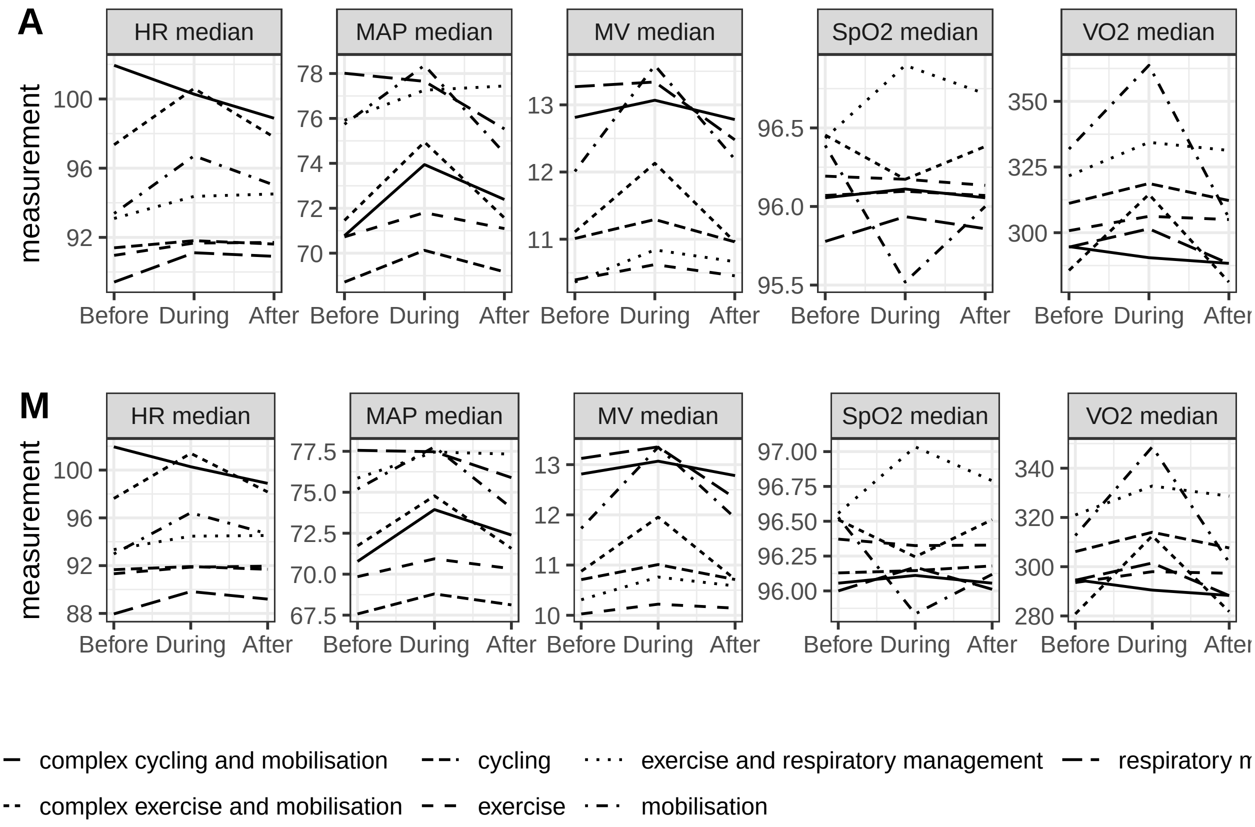

Tables 2 and 3

Note: We used Satterthwaite's method for calculating the degrees of freedom and t-statistics. In case of not reliable calculations we changed to Kenward-Roger's method (see *?summary.lmerTest*).

```
# Fill baseline information for repeated measures
data0 <- data %>% group_by(record_id) %>% mutate(age=first(age), sex=first(sex),
                                                  bmi=first(bmi))

# Setting reference levels
data0$Mob_level <- factor(data0$Mob_level, levels=c("in-bed", "edge-of-bed",
                                                     "out-of-bed"))
data0$Rx_modal <- factor(data0$Rx_modal, levels=c("passive", "mixed", "active"))
data0$Airway <- factor(data0$Airway, levels=c("none", "tracheostomy", "tube"))
data0$phtp_type <- factor(data0$phtp_type, levels=c("exercise", "cycling",
                          "mobilisation", "respiratory management",
                          "exercise and respiratory management",
                          "complex cycling and mobilisation",
                          "complex exercise and mobilisation"))
data0$sex <- factor(data0$sex, levels=c("male", "female"))


#
# VO2_md_during analysis
#
```

```r
vars_to_incl <- c("record_id", "VO2_md_during", "VO2_md_prior",  "VO2_CV_prior",
                  "age",  "bmi", "Mob_level", "Rx_duration", "Rx_modal",
                  "in_study_tm", "Rx_sofa", "relax", "vaso", "opia", "sed",
                  "Airway", "phtp_type","sex")

vo2_during.lme <- lmer(VO2_md_during ~ age+sex+bmi+Rx_sofa+Rx_duration+
                       in_study_tm+phtp_type+Rx_modal+Mob_level+Airway+opia+
                       vaso+sed+relax+VO2_md_prior+VO2_CV_prior+(1|record_id),
                       data = na.omit(data0[ ,vars_to_incl]))

summary(vo2_during.lme)
```

```
## Linear mixed model fit by REML. t-tests use Satterthwaite's method [
## lmerModLmerTest]
## Formula:
## VO2_md_during ~ age + sex + bmi + Rx_sofa + Rx_duration + in_study_tm +
##     phtp_type + Rx_modal + Mob_level + Airway + opia + vaso +
##     sed + relax + VO2_md_prior + VO2_CV_prior + (1 | record_id)
##    Data: na.omit(data0[, vars_to_incl])
##
## REML criterion at convergence: 2959.2
##
## Scaled residuals:
##     Min      1Q  Median      3Q     Max
## -3.8628 -0.4730 -0.0765  0.4060  7.5734
##
## Random effects:
##  Groups    Name        Variance Std.Dev.
##  record_id (Intercept)   32.73   5.721
##  Residual              1098.82  33.148
## Number of obs: 312, groups:  record_id, 94
##
## Fixed effects:
##                                               Estimate Std. Error        df
## (Intercept)                                  100.54452   25.08217 225.64351
## age                                           -0.54282    0.17588 177.67592
## sexfemale                                    -23.77775    4.60049  95.45915
## bmi                                            0.88049    0.48083 148.90954
## Rx_sofa                                        0.28747    0.55268 151.86082
## Rx_duration                                   -0.58611    0.25145 281.07188
## in_study_tm                                   -0.17629    0.20889 196.89023
## phtp_typecycling                               3.52194    5.42609 210.94755
## phtp_typemobilisation                         38.54227   27.49050 285.56227
## phtp_typerespiratory management                1.55943   33.97307 285.29556
## phtp_typeexercise and respiratory management  -0.04131    7.62332 284.56863
## phtp_typecomplex cycling and mobilisation     -7.50832   14.87956 286.43221
## phtp_typecomplex exercise and mobilisation    19.71483   24.70861 283.53651
## Rx_modalmixed                                  1.10582   10.28053 285.91436
## Rx_modalactive                                23.00698    5.69241 266.55349
## Mob_leveledge-of-bed                          -2.84978   26.37822 285.41037
## Mob_levelout-of-bed                            6.86557   28.55009 286.62070
## Airwaytracheostomy                             1.35809   13.80265 285.90118
## Airwaytube                                     0.84827   12.94948 285.60195
## opiayes                                       -7.43686    9.24730 246.35334
```

```
## vasoyes                                          -6.91561    4.76872 263.40674
## sedyes                                            4.88004    7.44530 269.18302
## relaxyes                                         -6.73058    5.31686 286.36210
## VO2_md_prior                                      0.82030    0.02438 265.87246
## VO2_CV_prior                                     -0.14001    0.05902 286.98021
##                                                t value Pr(>|t|)
## (Intercept)                                      4.009 8.30e-05 ***
## age                                             -3.086  0.00235 **
## sexfemale                                       -5.169 1.29e-06 ***
## bmi                                              1.831  0.06907 .
## Rx_sofa                                          0.520  0.60373
## Rx_duration                                     -2.331  0.02046 *
## in_study_tm                                     -0.844  0.39972
## phtp_typecycling                                 0.649  0.51700
## phtp_typemobilisation                            1.402  0.16199
## phtp_typerespiratory management                  0.046  0.96342
## phtp_typeexercise and respiratory management  -0.005  0.99568
## phtp_typecomplex cycling and mobilisation      -0.505  0.61422
## phtp_typecomplex exercise and mobilisation      0.798  0.42560
## Rx_modalmixed                                    0.108  0.91442
## Rx_modalactive                                   4.042 6.95e-05 ***
## Mob_leveledge-of-bed                            -0.108  0.91404
## Mob_levelout-of-bed                              0.240  0.81013
## Airwaytracheostomy                               0.098  0.92169
## Airwaytube                                       0.066  0.94782
## opiayes                                         -0.804  0.42205
## vasoyes                                         -1.450  0.14819
## sedyes                                           0.655  0.51274
## relaxyes                                        -1.266  0.20658
## VO2_md_prior                                    33.641  < 2e-16 ***
## VO2_CV_prior                                    -2.372  0.01834 *
## ---
## Signif. codes:  0 '***' 0.001 '**' 0.01 '*' 0.05 '.' 0.1 ' ' 1
```

```
### Test for overall category
### phtp_type
vo2_during.lme0 <- lmer(VO2_md_during ~ age+sex+bmi+Rx_sofa+Rx_duration+
                        in_study_tm+Rx_modal+Mob_level+Airway+opia+
                        vaso+sed+relax+VO2_md_prior+VO2_CV_prior+(1|record_id),
                        data = na.omit(data0[ ,vars_to_incl]))

anova(vo2_during.lme, vo2_during.lme0, test="LRT")
```

```
## Data: na.omit(data0[, vars_to_incl])
## Models:
## vo2_during.lme0: VO2_md_during ~ age + sex + bmi + Rx_sofa + Rx_duration + in_study_tm +
## vo2_during.lme0:     Rx_modal + Mob_level + Airway + opia + vaso + sed + relax +
## vo2_during.lme0:     VO2_md_prior + VO2_CV_prior + (1 | record_id)
## vo2_during.lme: VO2_md_during ~ age + sex + bmi + Rx_sofa + Rx_duration + in_study_tm +
## vo2_during.lme:     phtp_type + Rx_modal + Mob_level + Airway + opia + vaso +
## vo2_during.lme:     sed + relax + VO2_md_prior + VO2_CV_prior + (1 | record_id)
##                 npar    AIC    BIC  logLik deviance  Chisq Df Pr(>Chisq)
## vo2_during.lme0   21 3098.7 3177.3 -1528.4   3056.7
## vo2_during.lme    27 3106.3 3207.3 -1526.1   3052.3 4.4586  6     0.6149
```

```r
### Rx_modal
vo2_during.lme0 <- lmer(VO2_md_during ~ age+sex+bmi+Rx_sofa+Rx_duration+
                        in_study_tm+phtp_type+Mob_level+Airway+opia+
                        vaso+sed+relax+VO2_md_prior+VO2_CV_prior+(1|record_id),
                        data = na.omit(data0[ ,vars_to_incl]))

anova(vo2_during.lme, vo2_during.lme0, test="LRT")
```

```
## Data: na.omit(data0[, vars_to_incl])
## Models:
## vo2_during.lme0: VO2_md_during ~ age + sex + bmi + Rx_sofa + Rx_duration + in_study_tm +
## vo2_during.lme0:     phtp_type + Mob_level + Airway + opia + vaso + sed + relax +
## vo2_during.lme0:     VO2_md_prior + VO2_CV_prior + (1 | record_id)
## vo2_during.lme: VO2_md_during ~ age + sex + bmi + Rx_sofa + Rx_duration + in_study_tm +
## vo2_during.lme:     phtp_type + Rx_modal + Mob_level + Airway + opia + vaso +
## vo2_during.lme:     sed + relax + VO2_md_prior + VO2_CV_prior + (1 | record_id)
##                 npar    AIC    BIC  logLik deviance  Chisq Df Pr(>Chisq)
## vo2_during.lme0   25 3120.1 3213.7 -1535.0   3070.1
## vo2_during.lme    27 3106.3 3207.3 -1526.1   3052.3 17.835  2   0.000134 ***
## ---
## Signif. codes:  0 '***' 0.001 '**' 0.01 '*' 0.05 '.' 0.1 ' ' 1
```

```r
### Airway
vo2_during.lme0 <- lmer(VO2_md_during ~ age+sex+bmi+Rx_sofa+Rx_duration+
                        in_study_tm+phtp_type+Rx_modal+Mob_level+opia+
                        vaso+sed+relax+VO2_md_prior+VO2_CV_prior+(1|record_id),
                        data = na.omit(data0[ ,vars_to_incl]))

anova(vo2_during.lme, vo2_during.lme0, test="LRT")
```

```
## Data: na.omit(data0[, vars_to_incl])
## Models:
## vo2_during.lme0: VO2_md_during ~ age + sex + bmi + Rx_sofa + Rx_duration + in_study_tm +
## vo2_during.lme0:     phtp_type + Rx_modal + Mob_level + opia + vaso + sed + relax +
## vo2_during.lme0:     VO2_md_prior + VO2_CV_prior + (1 | record_id)
## vo2_during.lme: VO2_md_during ~ age + sex + bmi + Rx_sofa + Rx_duration + in_study_tm +
## vo2_during.lme:     phtp_type + Rx_modal + Mob_level + Airway + opia + vaso +
## vo2_during.lme:     sed + relax + VO2_md_prior + VO2_CV_prior + (1 | record_id)
##                 npar    AIC    BIC  logLik deviance  Chisq Df Pr(>Chisq)
## vo2_during.lme0   25 3102.3 3195.9 -1526.1   3052.3
## vo2_during.lme    27 3106.3 3207.3 -1526.1   3052.3 0.0244  2     0.9879
```

```r
### Mob_level
vo2_during.lme0 <- lmer(VO2_md_during ~ age+sex+bmi+Rx_sofa+Rx_duration+
                        in_study_tm+phtp_type+Rx_modal+Airway+opia+
                        vaso+sed+relax+VO2_md_prior+VO2_CV_prior+(1|record_id),
                        data = na.omit(data0[ ,vars_to_incl]))

anova(vo2_during.lme, vo2_during.lme0, test="LRT")
```

```
## Data: na.omit(data0[, vars_to_incl])
## Models:
## vo2_during.lme0: VO2_md_during ~ age + sex + bmi + Rx_sofa + Rx_duration + in_study_tm +
## vo2_during.lme0:     phtp_type + Rx_modal + Airway + opia + vaso + sed + relax +
## vo2_during.lme0:     VO2_md_prior + VO2_CV_prior + (1 | record_id)
```

```
## vo2_during.lme: VO2_md_during ~ age + sex + bmi + Rx_sofa + Rx_duration + in_study_tm +
## vo2_during.lme:     phtp_type + Rx_modal + Mob_level + Airway + opia + vaso +
## vo2_during.lme:     sed + relax + VO2_md_prior + VO2_CV_prior + (1 | record_id)
##                   npar    AIC    BIC  logLik deviance  Chisq Df Pr(>Chisq)
## vo2_during.lme0   25 3103.0 3196.6 -1526.5   3053.0
## vo2_during.lme    27 3106.3 3207.3 -1526.1   3052.3 0.7683  2      0.681
```

```
#
# VO2_md_after analysis
#

vars_to_incl <- c("record_id", "VO2_md_after", "VO2_md_prior", "VO2_CV_prior",
                  "age",  "bmi", "Mob_level", "Rx_duration", "in_study_tm",
                  "Rx_sofa", "relax", "Rx_modal",
                  "vaso", "opia", "sed", "Airway", "phtp_type","sex")

vo2_after.lme <- lmer(VO2_md_after ~ age+sex+bmi+Rx_sofa+Rx_duration+
                       in_study_tm+phtp_type+Rx_modal+Mob_level+Airway+opia+
                       vaso+sed+relax+VO2_md_prior+VO2_CV_prior+(1|record_id),
                       data = na.omit(data0[ ,vars_to_incl]))

summary(vo2_after.lme, ddf="Kenward-Roger")
```

```
## Linear mixed model fit by REML. t-tests use Kenward-Roger's method [
## lmerModLmerTest]
## Formula:
## VO2_md_after ~ age + sex + bmi + Rx_sofa + Rx_duration + in_study_tm +
##     phtp_type + Rx_modal + Mob_level + Airway + opia + vaso +
##     sed + relax + VO2_md_prior + VO2_CV_prior + (1 | record_id)
##    Data: na.omit(data0[, vars_to_incl])
##
## REML criterion at convergence: 2980.9
##
## Scaled residuals:
##     Min      1Q  Median      3Q     Max
## -7.4443 -0.4575 -0.1024  0.4680  3.0635
##
## Random effects:
##  Groups    Name        Variance Std.Dev.
##  record_id (Intercept)    0      0.00
##  Residual              1401     37.43
## Number of obs: 308, groups:  record_id, 94
##
## Fixed effects:
##                                              Estimate Std. Error        df
## (Intercept)                                  92.43142   28.37399 173.07527
## age                                          -0.30055    0.19477 124.93252
## sexfemale                                   -18.23025    5.04712  44.45501
## bmi                                           0.52133    0.52868  93.99485
## Rx_sofa                                      -0.32182    0.60959  90.81204
## Rx_duration                                  -0.28035    0.29883 273.29829
## in_study_tm                                   0.07657    0.24005 104.65320
## phtp_typecycling                             -2.37968    6.06630 149.63825
## phtp_typemobilisation                       -12.16702   31.54201 282.83425
## phtp_typerespiratory management             -15.98028   38.42097 282.98695
```

```
## phtp_typeexercise and respiratory management  -4.17066    8.66344 277.16956
## phtp_typecomplex cycling and mobilisation    -11.64470   16.74816 277.01453
## phtp_typecomplex exercise and mobilisation   -12.63182   27.90594 281.11508
## Rx_modalmixed                                 -6.55482   11.56459 276.78099
## Rx_modalactive                                11.97727    6.48011 213.09676
## Mob_leveledge-of-bed                          13.40723   29.83254 282.60794
## Mob_levelout-of-bed                          -40.92174   32.63171 282.62982
## Airwaytracheostomy                             4.87530   15.57126 272.50982
## Airwaytube                                     6.33764   14.58895 273.47573
## opiayes                                       -8.26615   10.39269 174.52126
## vasoyes                                      -11.78357    5.36786 213.18925
## sedyes                                        10.85527    8.67873 202.47307
## relaxyes                                     -10.12238    5.96967 279.45496
## VO2_md_prior                                   0.79322    0.02975 213.86209
## VO2_CV_prior                                  -0.10759    0.06658 282.31612
##                                              t value Pr(>|t|)
## (Intercept)                                    3.258 0.001352 **
## age                                           -1.543 0.125323
## sexfemale                                     -3.612 0.000769 ***
## bmi                                            0.986 0.326619
## Rx_sofa                                       -0.528 0.598837
## Rx_duration                                   -0.938 0.348988
## in_study_tm                                    0.319 0.750376
## phtp_typecycling                              -0.392 0.695410
## phtp_typemobilisation                         -0.386 0.699979
## phtp_typerespiratory management               -0.416 0.677779
## phtp_typeexercise and respiratory management  -0.481 0.630606
## phtp_typecomplex cycling and mobilisation     -0.695 0.487461
## phtp_typecomplex exercise and mobilisation    -0.453 0.651145
## Rx_modalmixed                                 -0.567 0.571309
## Rx_modalactive                                 1.848 0.065943 .
## Mob_leveledge-of-bed                           0.449 0.653476
## Mob_levelout-of-bed                           -1.254 0.210861
## Airwaytracheostomy                             0.313 0.754447
## Airwaytube                                     0.434 0.664331
## opiayes                                       -0.795 0.427473
## vasoyes                                       -2.195 0.029228 *
## sedyes                                         1.251 0.212454
## relaxyes                                      -1.696 0.091069 .
## VO2_md_prior                                  26.660  < 2e-16 ***
## VO2_CV_prior                                  -1.616 0.107227
## ---
## Signif. codes:  0 '***' 0.001 '**' 0.01 '*' 0.05 '.' 0.1 ' ' 1
## convergence code: 0
## boundary (singular) fit: see ?isSingular
```

```
### Test for overall category
### phtp_type
vo2_after.lme0 <- lmer(VO2_md_after ~ age+sex+bmi+Rx_sofa+Rx_duration+
                       in_study_tm+Rx_modal+Mob_level+Airway+opia+
                       vaso+sed+relax+VO2_md_prior+VO2_CV_prior+(1|record_id),
                       data = na.omit(data0[ ,vars_to_incl]))

anova(vo2_after.lme, vo2_after.lme0, test="LRT")
```

```
## Data: na.omit(data0[, vars_to_incl])
## Models:
## vo2_after.lme0: VO2_md_after ~ age + sex + bmi + Rx_sofa + Rx_duration + in_study_tm +
## vo2_after.lme0:     Rx_modal + Mob_level + Airway + opia + vaso + sed + relax +
## vo2_after.lme0:     VO2_md_prior + VO2_CV_prior + (1 | record_id)
## vo2_after.lme: VO2_md_after ~ age + sex + bmi + Rx_sofa + Rx_duration + in_study_tm +
## vo2_after.lme:     phtp_type + Rx_modal + Mob_level + Airway + opia + vaso +
## vo2_after.lme:     sed + relax + VO2_md_prior + VO2_CV_prior + (1 | record_id)
##                npar    AIC    BIC  logLik deviance  Chisq Df Pr(>Chisq)
## vo2_after.lme0   21 3122.5 3200.8 -1540.2   3080.5
## vo2_after.lme    27 3133.4 3234.1 -1539.7   3079.4 1.0928  6     0.9818
```

```r
### Rx_modal
vo2_after.lme0 <- lmer(VO2_md_after ~ age+sex+bmi+Rx_sofa+Rx_duration+
                       in_study_tm+phtp_type+Mob_level+Airway+opia+
                       vaso+sed+relax+VO2_md_prior+VO2_CV_prior+(1|record_id),
                       data = na.omit(data0[ ,vars_to_incl]))

anova(vo2_after.lme, vo2_after.lme0, test="LRT")
```

```
## Data: na.omit(data0[, vars_to_incl])
## Models:
## vo2_after.lme0: VO2_md_after ~ age + sex + bmi + Rx_sofa + Rx_duration + in_study_tm +
## vo2_after.lme0:     phtp_type + Mob_level + Airway + opia + vaso + sed + relax +
## vo2_after.lme0:     VO2_md_prior + VO2_CV_prior + (1 | record_id)
## vo2_after.lme: VO2_md_after ~ age + sex + bmi + Rx_sofa + Rx_duration + in_study_tm +
## vo2_after.lme:     phtp_type + Rx_modal + Mob_level + Airway + opia + vaso +
## vo2_after.lme:     sed + relax + VO2_md_prior + VO2_CV_prior + (1 | record_id)
##                npar    AIC    BIC  logLik deviance Chisq Df Pr(>Chisq)
## vo2_after.lme0   25 3134.1 3227.4 -1542.1   3084.1
## vo2_after.lme    27 3133.4 3234.1 -1539.7   3079.4 4.699  2    0.09542 .
## ---
## Signif. codes:  0 '***' 0.001 '**' 0.01 '*' 0.05 '.' 0.1 ' ' 1
```

```r
### Airway
vo2_after.lme0 <- lmer(VO2_md_after ~ age+sex+bmi+Rx_sofa+Rx_duration+
                       in_study_tm+phtp_type+Rx_modal+Mob_level+opia+
                       vaso+sed+relax+VO2_md_prior+VO2_CV_prior+(1|record_id),
                       data = na.omit(data0[ ,vars_to_incl]))

anova(vo2_after.lme, vo2_after.lme0, test="LRT")
```

```
## Data: na.omit(data0[, vars_to_incl])
## Models:
## vo2_after.lme0: VO2_md_after ~ age + sex + bmi + Rx_sofa + Rx_duration + in_study_tm +
## vo2_after.lme0:     phtp_type + Rx_modal + Mob_level + opia + vaso + sed + relax +
## vo2_after.lme0:     VO2_md_prior + VO2_CV_prior + (1 | record_id)
## vo2_after.lme: VO2_md_after ~ age + sex + bmi + Rx_sofa + Rx_duration + in_study_tm +
## vo2_after.lme:     phtp_type + Rx_modal + Mob_level + Airway + opia + vaso +
## vo2_after.lme:     sed + relax + VO2_md_prior + VO2_CV_prior + (1 | record_id)
##                npar    AIC    BIC  logLik deviance Chisq Df Pr(>Chisq)
## vo2_after.lme0   25 3129.7 3222.9 -1539.8   3079.7
## vo2_after.lme    27 3133.4 3234.1 -1539.7   3079.4 0.243  2     0.8856
```

```r
### Mob_level
vo2_after.lme0 <- lmer(VO2_md_after ~ age+sex+bmi+Rx_sofa+Rx_duration+
```

```r
                        in_study_tm+phtp_type+Rx_modal+Airway+opia+
                        vaso+sed+relax+VO2_md_prior+VO2_CV_prior+(1|record_id),
                        data = na.omit(data0[ ,vars_to_incl]))

anova(vo2_after.lme, vo2_after.lme0, test="LRT")
```

```
## Data: na.omit(data0[, vars_to_incl])
## Models:
## vo2_after.lme0: VO2_md_after ~ age + sex + bmi + Rx_sofa + Rx_duration + in_study_tm +
## vo2_after.lme0:     phtp_type + Rx_modal + Airway + opia + vaso + sed + relax +
## vo2_after.lme0:     VO2_md_prior + VO2_CV_prior + (1 | record_id)
## vo2_after.lme: VO2_md_after ~ age + sex + bmi + Rx_sofa + Rx_duration + in_study_tm +
## vo2_after.lme:     phtp_type + Rx_modal + Mob_level + Airway + opia + vaso +
## vo2_after.lme:     sed + relax + VO2_md_prior + VO2_CV_prior + (1 | record_id)
##                npar    AIC    BIC  logLik deviance  Chisq Df Pr(>Chisq)
## vo2_after.lme0   25 3144.1 3237.3 -1547.0   3094.1
## vo2_after.lme    27 3133.4 3234.1 -1539.7   3079.4 14.656  2  0.0006567 ***
## ---
## Signif. codes:  0 '***' 0.001 '**' 0.01 '*' 0.05 '.' 0.1 ' ' 1
```

```r
#
# MV_during analysis
#

vars_to_incl <- c("record_id", "MV_md_during", "MV_md_prior", "MV_CV_prior",
                  "age",  "bmi", "Mob_level", "Rx_duration", "in_study_tm",
                  "Rx_sofa", "relax", "Rx_modal",
                  "vaso", "opia", "sed", "Airway", "phtp_type","sex")

mv_during.lme <- lmer(MV_md_during ~ age+sex+bmi+Rx_sofa+Rx_duration+
                        in_study_tm+phtp_type+Rx_modal+Mob_level+Airway+opia+
                        vaso+sed+relax+MV_md_prior+MV_CV_prior+(1|record_id),
                        data = na.omit(data0[ ,vars_to_incl]))

summary(mv_during.lme)
```

```
## Linear mixed model fit by REML. t-tests use Satterthwaite's method [
## lmerModLmerTest]
## Formula:
## MV_md_during ~ age + sex + bmi + Rx_sofa + Rx_duration + in_study_tm +
##     phtp_type + Rx_modal + Mob_level + Airway + opia + vaso +
##     sed + relax + MV_md_prior + MV_CV_prior + (1 | record_id)
##    Data: na.omit(data0[, vars_to_incl])
##
## REML criterion at convergence: 1473.5
##
## Scaled residuals:
##     Min      1Q  Median      3Q     Max
## -3.1223 -0.5120 -0.1122  0.3957  5.4547
##
## Random effects:
##  Groups    Name        Variance Std.Dev.
##  record_id (Intercept) 0.1149   0.339
##  Residual              1.3772   1.174
## Number of obs: 442, groups:  record_id, 103
```

```
##
## Fixed effects:
##                                              Estimate Std. Error         df
## (Intercept)                                  2.352150   0.792499 224.925678
## age                                         -0.001686   0.005878 160.398701
## sexfemale                                   -0.416409   0.160741  86.647477
## bmi                                         -0.010288   0.015573 121.695401
## Rx_sofa                                      0.014995   0.017778 191.605899
## Rx_duration                                 -0.017626   0.006987 414.992366
## in_study_tm                                  0.001218   0.006801 357.669211
## phtp_typecycling                             0.150560   0.169857 346.272657
## phtp_typemobilisation                        0.789031   0.599071 415.764781
## phtp_typerespiratory management              0.356544   0.386903 415.288861
## phtp_typeexercise and respiratory management -0.020032   0.233262 416.706393
## phtp_typecomplex cycling and mobilisation    0.190285   0.467281 414.268028
## phtp_typecomplex exercise and mobilisation   0.235507   0.571937 416.236726
## Rx_modalmixed                                0.530823   0.311311 416.885724
## Rx_modalactive                               0.715445   0.168125 399.220225
## Mob_leveledge-of-bed                         0.743366   0.598545 416.390482
## Mob_levelout-of-bed                          0.356191   0.625857 416.997503
## Airwaytracheostomy                           0.295539   0.333666 413.832743
## Airwaytube                                   0.048629   0.317399 411.447655
## opiayes                                      0.115078   0.289955 410.554817
## vasoyes                                     -0.133733   0.145969 402.894446
## sedyes                                       0.037035   0.196965 408.605460
## relaxyes                                    -0.205089   0.162568 416.802883
## MV_md_prior                                  0.872473   0.022678 307.216508
## MV_CV_prior                                 -0.009332   0.002989 416.942627
##                                             t value Pr(>|t|)
## (Intercept)                                   2.968  0.00332 **
## age                                          -0.287  0.77463
## sexfemale                                    -2.591  0.01124 *
## bmi                                          -0.661  0.51010
## Rx_sofa                                       0.843  0.40003
## Rx_duration                                  -2.523  0.01201 *
## in_study_tm                                   0.179  0.85796
## phtp_typecycling                              0.886  0.37602
## phtp_typemobilisation                         1.317  0.18853
## phtp_typerespiratory management               0.922  0.35731
## phtp_typeexercise and respiratory management -0.086  0.93160
## phtp_typecomplex cycling and mobilisation     0.407  0.68406
## phtp_typecomplex exercise and mobilisation    0.412  0.68072
## Rx_modalmixed                                 1.705  0.08892 .
## Rx_modalactive                                4.255  2.6e-05 ***
## Mob_leveledge-of-bed                          1.242  0.21495
## Mob_levelout-of-bed                           0.569  0.56958
## Airwaytracheostomy                            0.886  0.37628
## Airwaytube                                    0.153  0.87831
## opiayes                                       0.397  0.69166
## vasoyes                                      -0.916  0.36012
## sedyes                                        0.188  0.85095
## relaxyes                                     -1.262  0.20781
## MV_md_prior                                  38.473  < 2e-16 ***
## MV_CV_prior                                  -3.122  0.00192 **
```

```
## ---
## Signif. codes:  0 '***' 0.001 '**' 0.01 '*' 0.05 '.' 0.1 ' ' 1
```

```r
### Test for overall category
### phtp_type
mv_during.lme0 <- lmer(MV_md_during ~ age+sex+bmi+Rx_sofa+Rx_duration+
                       in_study_tm+Rx_modal+Mob_level+Airway+opia+
                       vaso+sed+relax+MV_md_prior+MV_CV_prior+(1|record_id),
                       data = na.omit(data0[ ,vars_to_incl]))

anova(mv_during.lme, mv_during.lme0, test="LRT")
```

```
## Data: na.omit(data0[, vars_to_incl])
## Models:
## mv_during.lme0: MV_md_during ~ age + sex + bmi + Rx_sofa + Rx_duration + in_study_tm +
## mv_during.lme0:     Rx_modal + Mob_level + Airway + opia + vaso + sed + relax +
## mv_during.lme0:     MV_md_prior + MV_CV_prior + (1 | record_id)
## mv_during.lme: MV_md_during ~ age + sex + bmi + Rx_sofa + Rx_duration + in_study_tm +
## mv_during.lme:     phtp_type + Rx_modal + Mob_level + Airway + opia + vaso +
## mv_during.lme:     sed + relax + MV_md_prior + MV_CV_prior + (1 | record_id)
##                npar    AIC    BIC  logLik deviance  Chisq Df Pr(>Chisq)
## mv_during.lme0   21 1445.4 1531.3 -701.71   1403.4
## mv_during.lme    27 1451.5 1562.0 -698.76   1397.5 5.9018  6     0.4343
```

```r
### Rx_modal
mv_during.lme0 <- lmer(MV_md_during ~ age+sex+bmi+Rx_sofa+Rx_duration+
                       in_study_tm+phtp_type+Mob_level+Airway+opia+
                       vaso+sed+relax+MV_md_prior+MV_CV_prior+(1|record_id),
                       data = na.omit(data0[ ,vars_to_incl]))

anova(mv_during.lme, mv_during.lme0, test="LRT")
```

```
## Data: na.omit(data0[, vars_to_incl])
## Models:
## mv_during.lme0: MV_md_during ~ age + sex + bmi + Rx_sofa + Rx_duration + in_study_tm +
## mv_during.lme0:     phtp_type + Mob_level + Airway + opia + vaso + sed + relax +
## mv_during.lme0:     MV_md_prior + MV_CV_prior + (1 | record_id)
## mv_during.lme: MV_md_during ~ age + sex + bmi + Rx_sofa + Rx_duration + in_study_tm +
## mv_during.lme:     phtp_type + Rx_modal + Mob_level + Airway + opia + vaso +
## mv_during.lme:     sed + relax + MV_md_prior + MV_CV_prior + (1 | record_id)
##                npar    AIC    BIC  logLik deviance  Chisq Df Pr(>Chisq)
## mv_during.lme0   25 1467.5 1569.8 -708.76   1417.5
## mv_during.lme    27 1451.5 1562.0 -698.76   1397.5 19.993  2  4.556e-05 ***
## ---
## Signif. codes:  0 '***' 0.001 '**' 0.01 '*' 0.05 '.' 0.1 ' ' 1
```

```r
### Airway
mv_during.lme0 <- lmer(MV_md_during ~ age+sex+bmi+Rx_sofa+Rx_duration+
                       in_study_tm+phtp_type+Rx_modal+Mob_level+opia+
                       vaso+sed+relax+MV_md_prior+MV_CV_prior+(1|record_id),
                       data = na.omit(data0[ ,vars_to_incl]))


anova(mv_during.lme, mv_during.lme0, test="LRT")
```

```
## Data: na.omit(data0[, vars_to_incl])
```

```
## Models:
## mv_during.lme0: MV_md_during ~ age + sex + bmi + Rx_sofa + Rx_duration + in_study_tm +
## mv_during.lme0:     phtp_type + Rx_modal + Mob_level + opia + vaso + sed + relax +
## mv_during.lme0:     MV_md_prior + MV_CV_prior + (1 | record_id)
## mv_during.lme: MV_md_during ~ age + sex + bmi + Rx_sofa + Rx_duration + in_study_tm +
## mv_during.lme:     phtp_type + Rx_modal + Mob_level + Airway + opia + vaso +
## mv_during.lme:     sed + relax + MV_md_prior + MV_CV_prior + (1 | record_id)
##                npar    AIC    BIC  logLik deviance  Chisq Df Pr(>Chisq)
## mv_during.lme0   25 1449.5 1551.7 -699.73   1399.5
## mv_during.lme    27 1451.5 1562.0 -698.76   1397.5 1.9392  2     0.3792
```

```
### Mob_level
mv_during.lme0 <- lmer(MV_md_during ~ age+sex+bmi+Rx_sofa+Rx_duration+
                       in_study_tm+phtp_type+Rx_modal+Airway+opia+
                       vaso+sed+relax+MV_md_prior+MV_CV_prior+(1|record_id),
                       data = na.omit(data0[ ,vars_to_incl]))

anova(mv_during.lme, mv_during.lme0, test="LRT")
```

```
## Data: na.omit(data0[, vars_to_incl])
## Models:
## mv_during.lme0: MV_md_during ~ age + sex + bmi + Rx_sofa + Rx_duration + in_study_tm +
## mv_during.lme0:     phtp_type + Rx_modal + Airway + opia + vaso + sed + relax +
## mv_during.lme0:     MV_md_prior + MV_CV_prior + (1 | record_id)
## mv_during.lme: MV_md_during ~ age + sex + bmi + Rx_sofa + Rx_duration + in_study_tm +
## mv_during.lme:     phtp_type + Rx_modal + Mob_level + Airway + opia + vaso +
## mv_during.lme:     sed + relax + MV_md_prior + MV_CV_prior + (1 | record_id)
##                npar    AIC    BIC  logLik deviance  Chisq Df Pr(>Chisq)
## mv_during.lme0   25 1450.0 1552.2 -699.98   1400.0
## mv_during.lme    27 1451.5 1562.0 -698.76   1397.5 2.4494  2     0.2938
```

```
#
# MV_after analysis
#

vars_to_incl <- c("record_id", "MV_md_after", "MV_md_prior", "MV_CV_prior",
                  "age",  "bmi", "Mob_level", "Rx_duration", "in_study_tm",
                  "Rx_sofa", "relax", "Rx_modal",
                  "vaso", "opia", "sed", "Airway", "phtp_type","sex")

mv_after.lme <- lmer(MV_md_after ~ age+sex+bmi+Rx_sofa+Rx_duration+
                       in_study_tm+phtp_type+Rx_modal+Mob_level+Airway+opia+
                       vaso+sed+relax+MV_md_prior+MV_CV_prior+(1|record_id),
                       data = na.omit(data0[ ,vars_to_incl]))

summary(mv_after.lme, ddf="Kenward-Roger")
```

```
## Linear mixed model fit by REML. t-tests use Kenward-Roger's method [
## lmerModLmerTest]
## Formula: MV_md_after ~ age + sex + bmi + Rx_sofa + Rx_duration + in_study_tm +
##     phtp_type + Rx_modal + Mob_level + Airway + opia + vaso +
##     sed + relax + MV_md_prior + MV_CV_prior + (1 | record_id)
##    Data: na.omit(data0[, vars_to_incl])
##
## REML criterion at convergence: 1529.2
```

```
##
## Scaled residuals:
##     Min      1Q  Median      3Q     Max
## -5.3362 -0.5503 -0.0915  0.5009  4.9594
##
## Random effects:
##  Groups    Name        Variance Std.Dev.
##  record_id (Intercept) 0.000    0.000
##  Residual              1.736    1.318
## Number of obs: 437, groups:  record_id, 103
##
## Fixed effects:
##                                              Estimate Std. Error         df
## (Intercept)                                 1.242e+00  8.194e-01  1.444e+02
## age                                         2.488e-03  5.848e-03  1.586e+02
## sexfemale                                  -2.935e-01  1.511e-01  3.891e+01
## bmi                                        -1.683e-02  1.518e-02  8.145e+01
## Rx_sofa                                    -8.438e-03  1.792e-02  1.215e+02
## Rx_duration                                -1.594e-04  7.731e-03  3.996e+02
## in_study_tm                                -3.120e-03  7.316e-03  1.460e+02
## phtp_typecycling                           -3.862e-02  1.796e-01  2.761e+02
## phtp_typemobilisation                       1.157e+00  6.621e-01  4.030e+02
## phtp_typerespiratory management            -8.058e-01  4.477e-01  3.026e+02
## phtp_typeexercise and respiratory management  6.901e-02  2.553e-01  4.086e+02
## phtp_typecomplex cycling and mobilisation     1.767e-01  5.077e-01  3.848e+02
## phtp_typecomplex exercise and mobilisation    1.808e-01  6.276e-01  4.096e+02
## Rx_modalmixed                              -4.272e-01  3.414e-01  4.085e+02
## Rx_modalactive                              1.917e-01  1.841e-01  3.251e+02
## Mob_leveledge-of-bed                       -3.855e-01  6.578e-01  3.955e+02
## Mob_levelout-of-bed                        -1.176e+00  6.890e-01  3.732e+02
## Airwaytracheostomy                          2.956e-01  3.709e-01  3.641e+02
## Airwaytube                                  1.817e-01  3.534e-01  3.726e+02
## opiayes                                     3.487e-01  3.255e-01  2.729e+02
## vasoyes                                    -2.824e-01  1.584e-01  3.631e+02
## sedyes                                      2.417e-01  2.194e-01  2.740e+02
## relaxyes                                   -6.336e-03  1.778e-01  3.928e+02
## MV_md_prior                                 9.106e-01  2.373e-02  1.902e+02
## MV_CV_prior                                -8.551e-03  3.316e-03  4.078e+02
##                                            t value Pr(>|t|)
## (Intercept)                                  1.515   0.1319
## age                                          0.425   0.6711
## sexfemale                                   -1.942   0.0594 .
## bmi                                         -1.109   0.2708
## Rx_sofa                                     -0.471   0.6385
## Rx_duration                                 -0.021   0.9836
## in_study_tm                                 -0.426   0.6704
## phtp_typecycling                            -0.215   0.8299
## phtp_typemobilisation                        1.748   0.0813 .
## phtp_typerespiratory management             -1.800   0.0729 .
## phtp_typeexercise and respiratory management   0.270   0.7871
## phtp_typecomplex cycling and mobilisation      0.348   0.7280
## phtp_typecomplex exercise and mobilisation     0.288   0.7734
## Rx_modalmixed                               -1.251   0.2116
## Rx_modalactive                               1.041   0.2986
```

```
## Mob_leveledge-of-bed                              -0.586   0.5581
## Mob_levelout-of-bed                               -1.706   0.0888 .
## Airwaytracheostomy                                 0.797   0.4261
## Airwaytube                                         0.514   0.6074
## opiayes                                            1.071   0.2850
## vasoyes                                           -1.783   0.0754 .
## sedyes                                             1.102   0.2715
## relaxyes                                          -0.036   0.9716
## MV_md_prior                                       38.378   <2e-16 ***
## MV_CV_prior                                       -2.579   0.0103 *
## ---
## Signif. codes:  0 '***' 0.001 '**' 0.01 '*' 0.05 '.' 0.1 ' ' 1
## convergence code: 0
## boundary (singular) fit: see ?isSingular
```

```
### Test for overall category
### phtp_type
mv_after.lme0 <- lmer(MV_md_after ~ age+sex+bmi+Rx_sofa+Rx_duration+
                       in_study_tm+Rx_modal+Mob_level+Airway+opia+
                       vaso+sed+relax+MV_md_prior+MV_CV_prior+(1|record_id),
                       data = na.omit(data0[ ,vars_to_incl]))

anova(mv_after.lme, mv_after.lme0, test="LRT")
```

```
## Data: na.omit(data0[, vars_to_incl])
## Models:
## mv_after.lme0: MV_md_after ~ age + sex + bmi + Rx_sofa + Rx_duration + in_study_tm +
## mv_after.lme0:     Rx_modal + Mob_level + Airway + opia + vaso + sed + relax +
## mv_after.lme0:     MV_md_prior + MV_CV_prior + (1 | record_id)
## mv_after.lme: MV_md_after ~ age + sex + bmi + Rx_sofa + Rx_duration + in_study_tm +
## mv_after.lme:     phtp_type + Rx_modal + Mob_level + Airway + opia + vaso +
## mv_after.lme:     sed + relax + MV_md_prior + MV_CV_prior + (1 | record_id)
##               npar    AIC    BIC  logLik deviance  Chisq Df Pr(>Chisq)
## mv_after.lme0   21 1510.1 1595.8 -734.06   1468.1
## mv_after.lme    27 1509.4 1619.6 -727.71   1455.4 12.697  6    0.04811 *
## ---
## Signif. codes:  0 '***' 0.001 '**' 0.01 '*' 0.05 '.' 0.1 ' ' 1
```

```
### Rx_modal
mv_after.lme0 <- lmer(MV_md_after ~ age+sex+bmi+Rx_sofa+Rx_duration+
                       in_study_tm+phtp_type+Mob_level+Airway+opia+
                       vaso+sed+relax+MV_md_prior+MV_CV_prior+(1|record_id),
                       data = na.omit(data0[ ,vars_to_incl]))

anova(mv_after.lme, mv_after.lme0, test="LRT")
```

```
## Data: na.omit(data0[, vars_to_incl])
## Models:
## mv_after.lme0: MV_md_after ~ age + sex + bmi + Rx_sofa + Rx_duration + in_study_tm +
## mv_after.lme0:     phtp_type + Mob_level + Airway + opia + vaso + sed + relax +
## mv_after.lme0:     MV_md_prior + MV_CV_prior + (1 | record_id)
## mv_after.lme: MV_md_after ~ age + sex + bmi + Rx_sofa + Rx_duration + in_study_tm +
## mv_after.lme:     phtp_type + Rx_modal + Mob_level + Airway + opia + vaso +
## mv_after.lme:     sed + relax + MV_md_prior + MV_CV_prior + (1 | record_id)
##               npar    AIC    BIC  logLik deviance Chisq Df Pr(>Chisq)
## mv_after.lme0   25 1508.8 1610.8 -729.42   1458.8
```

```
## mv_after.lme    27 1509.4 1619.6 -727.71   1455.4 3.421  2     0.1808
```

```r
### Airway
mv_after.lme0 <- lmer(MV_md_after ~ age+sex+bmi+Rx_sofa+Rx_duration+
                      in_study_tm+phtp_type+Rx_modal+Mob_level+opia+
                      vaso+sed+relax+MV_md_prior+MV_CV_prior+(1|record_id),
                      data = na.omit(data0[ ,vars_to_incl]))

anova(mv_after.lme, mv_after.lme0, test="LRT")
```

```
## Data: na.omit(data0[, vars_to_incl])
## Models:
## mv_after.lme0: MV_md_after ~ age + sex + bmi + Rx_sofa + Rx_duration + in_study_tm +
## mv_after.lme0:     phtp_type + Rx_modal + Mob_level + opia + vaso + sed + relax +
## mv_after.lme0:     MV_md_prior + MV_CV_prior + (1 | record_id)
## mv_after.lme: MV_md_after ~ age + sex + bmi + Rx_sofa + Rx_duration + in_study_tm +
## mv_after.lme:     phtp_type + Rx_modal + Mob_level + Airway + opia + vaso +
## mv_after.lme:     sed + relax + MV_md_prior + MV_CV_prior + (1 | record_id)
##               npar    AIC    BIC  logLik deviance  Chisq Df Pr(>Chisq)
## mv_after.lme0   25 1506.2 1608.2 -728.10   1456.2
## mv_after.lme    27 1509.4 1619.6 -727.71   1455.4 0.7766  2     0.6782
```

```r
### Mob_level
mv_after.lme0 <- lmer(MV_md_after ~ age+sex+bmi+Rx_sofa+Rx_duration+
                      in_study_tm+phtp_type+Rx_modal+Airway+opia+
                      vaso+sed+relax+MV_md_prior+MV_CV_prior+(1|record_id),
                      data = na.omit(data0[ ,vars_to_incl]))

anova(mv_after.lme, mv_after.lme0, test="LRT")
```

```
## Data: na.omit(data0[, vars_to_incl])
## Models:
## mv_after.lme0: MV_md_after ~ age + sex + bmi + Rx_sofa + Rx_duration + in_study_tm +
## mv_after.lme0:     phtp_type + Rx_modal + Airway + opia + vaso + sed + relax +
## mv_after.lme0:     MV_md_prior + MV_CV_prior + (1 | record_id)
## mv_after.lme: MV_md_after ~ age + sex + bmi + Rx_sofa + Rx_duration + in_study_tm +
## mv_after.lme:     phtp_type + Rx_modal + Mob_level + Airway + opia + vaso +
## mv_after.lme:     sed + relax + MV_md_prior + MV_CV_prior + (1 | record_id)
##               npar    AIC    BIC  logLik deviance Chisq Df Pr(>Chisq)
## mv_after.lme0   25 1511.4 1613.4 -730.71   1461.4
## mv_after.lme    27 1509.4 1619.6 -727.71   1455.4 6.009  2    0.04956 *
## ---
## Signif. codes:  0 '***' 0.001 '**' 0.01 '*' 0.05 '.' 0.1 ' ' 1
```

```r
#### MAP_md_during

vars_to_incl <- c("record_id", "MAP_md_during", "MAP_md_prior", "MAP_CV_prior",
                  "age",  "bmi","Mob_level", "Rx_duration", "in_study_tm",
                  "Rx_sofa", "relax", "Rx_modal",
                  "vaso", "opia", "sed", "Airway", "phtp_type","sex")

map_during.lme <- lmer(MAP_md_during ~ age+sex+bmi+Rx_sofa+Rx_duration+
                      in_study_tm+phtp_type+Rx_modal+Mob_level+Airway+opia+
                      vaso+sed+relax+MAP_md_prior+MAP_CV_prior+(1|record_id),
                      data = na.omit(data0[ ,vars_to_incl]))
```

```
summary(map_during.lme, ddf="Kenward-Roger")
```

```
## Linear mixed model fit by REML. t-tests use Kenward-Roger's method [
## lmerModLmerTest]
## Formula:
## MAP_md_during ~ age + sex + bmi + Rx_sofa + Rx_duration + in_study_tm +
##     phtp_type + Rx_modal + Mob_level + Airway + opia + vaso +
##     sed + relax + MAP_md_prior + MAP_CV_prior + (1 | record_id)
##    Data: na.omit(data0[, vars_to_incl])
##
## REML criterion at convergence: 3334.6
##
## Scaled residuals:
##     Min      1Q  Median      3Q     Max
## -3.8825 -0.5468 -0.0561  0.5768  2.7734
##
## Random effects:
##  Groups    Name        Variance Std.Dev.
##  record_id (Intercept)  0.00    0.00
##  Residual              30.47    5.52
## Number of obs: 535, groups:  record_id, 107
##
## Fixed effects:
##                                              Estimate Std. Error         df
## (Intercept)                                  7.981983   3.172168 154.428978
## age                                          0.009912   0.021390 158.997788
## sexfemale                                    1.032194   0.536949  37.353643
## bmi                                          0.035902   0.055000 103.358821
## Rx_sofa                                     -0.022871   0.067403 105.333969
## Rx_duration                                 -0.063997   0.026831 506.572263
## in_study_tm                                  0.025416   0.023545  75.833387
## phtp_typecycling                             0.447993   0.713314 342.563864
## phtp_typemobilisation                        0.281308   2.545358 503.452967
## phtp_typerespiratory management              1.834653   1.109953 475.634580
## phtp_typeexercise and respiratory management  0.252862   0.924992 480.599258
## phtp_typecomplex cycling and mobilisation    1.981219   2.032479 487.650208
## phtp_typecomplex exercise and mobilisation   1.276731   2.455515 509.408348
## Rx_modalmixed                                2.881539   1.342412 508.896904
## Rx_modalactive                               1.409478   0.661974 350.790075
## Mob_leveledge-of-bed                         0.884308   2.509041 506.772254
## Mob_levelout-of-bed                          0.813743   2.653707 500.892138
## Airwaytracheostomy                           0.941096   0.945532 251.214422
## Airwaytube                                   1.142376   0.852701 478.456243
## opiayes                                      0.615129   1.048268 357.613000
## vasoyes                                     -1.791808   0.590600 474.785564
## sedyes                                       1.317042   0.705482 396.498882
## relaxyes                                    -0.372314   0.693483 478.287222
## MAP_md_prior                                 0.867401   0.021322 223.410268
## MAP_CV_prior                                -0.005822   0.008964 440.130105
##                                             t value Pr(>|t|)
## (Intercept)                                   2.516  0.01288 *
## age                                           0.463  0.64370
## sexfemale                                     1.922  0.06222 .
## bmi                                           0.653  0.51536
```

```
## Rx_sofa                                        -0.339  0.73504
## Rx_duration                                    -2.385  0.01744 *
## in_study_tm                                     1.079  0.28380
## phtp_typecycling                                0.628  0.53039
## phtp_typemobilisation                           0.111  0.91204
## phtp_typerespiratory management                 1.653  0.09901 .
## phtp_typeexercise and respiratory management    0.273  0.78469
## phtp_typecomplex cycling and mobilisation       0.975  0.33015
## phtp_typecomplex exercise and mobilisation      0.520  0.60333
## Rx_modalmixed                                   2.147  0.03230 *
## Rx_modalactive                                  2.129  0.03393 *
## Mob_leveledge-of-bed                            0.352  0.72465
## Mob_levelout-of-bed                             0.307  0.75924
## Airwaytracheostomy                              0.995  0.32054
## Airwaytube                                      1.340  0.18097
## opiayes                                         0.587  0.55771
## vasoyes                                        -3.034  0.00255 **
## sedyes                                          1.867  0.06266 .
## relaxyes                                       -0.537  0.59160
## MAP_md_prior                                   40.681  < 2e-16 ***
## MAP_CV_prior                                   -0.649  0.51637
## ---
## Signif. codes:  0 '***' 0.001 '**' 0.01 '*' 0.05 '.' 0.1 ' ' 1
## convergence code: 0
## boundary (singular) fit: see ?isSingular
```

```r
### Test for overall category
### phtp_type
map_during.lme0 <- lmer(MAP_md_during ~ age+sex+bmi+Rx_sofa+Rx_duration+
                        in_study_tm+Rx_modal+Mob_level+Airway+opia+
                        vaso+sed+relax+MAP_md_prior+MAP_CV_prior+(1|record_id),
                        data = na.omit(data0[ ,vars_to_incl]))

anova(map_during.lme, map_during.lme0, test="LRT")
```

```
## Data: na.omit(data0[, vars_to_incl])
## Models:
## map_during.lme0: MAP_md_during ~ age + sex + bmi + Rx_sofa + Rx_duration + in_study_tm +
## map_during.lme0:     Rx_modal + Mob_level + Airway + opia + vaso + sed + relax +
## map_during.lme0:     MAP_md_prior + MAP_CV_prior + (1 | record_id)
## map_during.lme: MAP_md_during ~ age + sex + bmi + Rx_sofa + Rx_duration + in_study_tm +
## map_during.lme:     phtp_type + Rx_modal + Mob_level + Airway + opia + vaso +
## map_during.lme:     sed + relax + MAP_md_prior + MAP_CV_prior + (1 | record_id)
##                 npar    AIC    BIC  logLik deviance  Chisq Df Pr(>Chisq)
## map_during.lme0   21 3366.9 3456.9 -1662.5   3324.9
## map_during.lme    27 3374.6 3490.2 -1660.3   3320.6 4.3692  6     0.6268
```

```r
### Rx_modal
map_during.lme0 <- lmer(MAP_md_during ~ age+sex+bmi+Rx_sofa+Rx_duration+
                        in_study_tm+phtp_type+Mob_level+Airway+opia+
                        vaso+sed+relax+MAP_md_prior+MAP_CV_prior+(1|record_id),
                        data = na.omit(data0[ ,vars_to_incl]))

anova(map_during.lme, map_during.lme0, test="LRT")
```

```
## Data: na.omit(data0[, vars_to_incl])
## Models:
## map_during.lme0: MAP_md_during ~ age + sex + bmi + Rx_sofa + Rx_duration + in_study_tm +
## map_during.lme0:     phtp_type + Mob_level + Airway + opia + vaso + sed + relax +
## map_during.lme0:     MAP_md_prior + MAP_CV_prior + (1 | record_id)
## map_during.lme: MAP_md_during ~ age + sex + bmi + Rx_sofa + Rx_duration + in_study_tm +
## map_during.lme:     phtp_type + Rx_modal + Mob_level + Airway + opia + vaso +
## map_during.lme:     sed + relax + MAP_md_prior + MAP_CV_prior + (1 | record_id)
##                 npar    AIC    BIC  logLik deviance  Chisq Df Pr(>Chisq)
## map_during.lme0   25 3378.9 3485.9 -1664.4   3328.9
## map_during.lme    27 3374.6 3490.2 -1660.3   3320.6 8.3088  2    0.01569 *
## ---
## Signif. codes:  0 '***' 0.001 '**' 0.01 '*' 0.05 '.' 0.1 ' ' 1
```

```
### Airway
map_during.lme0 <- lmer(MAP_md_during ~ age+sex+bmi+Rx_sofa+Rx_duration+
                        in_study_tm+phtp_type+Rx_modal+Mob_level+opia+
                        vaso+sed+relax+MAP_md_prior+MAP_CV_prior+(1|record_id),
                        data = na.omit(data0[ ,vars_to_incl]))

anova(map_during.lme, map_during.lme0, test="LRT")
```

```
## Data: na.omit(data0[, vars_to_incl])
## Models:
## map_during.lme0: MAP_md_during ~ age + sex + bmi + Rx_sofa + Rx_duration + in_study_tm +
## map_during.lme0:     phtp_type + Rx_modal + Mob_level + opia + vaso + sed + relax +
## map_during.lme0:     MAP_md_prior + MAP_CV_prior + (1 | record_id)
## map_during.lme: MAP_md_during ~ age + sex + bmi + Rx_sofa + Rx_duration + in_study_tm +
## map_during.lme:     phtp_type + Rx_modal + Mob_level + Airway + opia + vaso +
## map_during.lme:     sed + relax + MAP_md_prior + MAP_CV_prior + (1 | record_id)
##                 npar    AIC    BIC  logLik deviance  Chisq Df Pr(>Chisq)
## map_during.lme0   25 3372.5 3479.6 -1661.2   3322.5
## map_during.lme    27 3374.6 3490.2 -1660.3   3320.6 1.9253  2     0.3819
```

```
### Mob_level
map_during.lme0 <- lmer(MAP_md_during ~ age+sex+bmi+Rx_sofa+Rx_duration+
                        in_study_tm+phtp_type+Rx_modal+Airway+opia+
                        vaso+sed+relax+MAP_md_prior+MAP_CV_prior+(1|record_id),
                        data = na.omit(data0[ ,vars_to_incl]))

anova(map_during.lme, map_during.lme0, test="LRT")
```

```
## Data: na.omit(data0[, vars_to_incl])
## Models:
## map_during.lme0: MAP_md_during ~ age + sex + bmi + Rx_sofa + Rx_duration + in_study_tm +
## map_during.lme0:     phtp_type + Rx_modal + Airway + opia + vaso + sed + relax +
## map_during.lme0:     MAP_md_prior + MAP_CV_prior + (1 | record_id)
## map_during.lme: MAP_md_during ~ age + sex + bmi + Rx_sofa + Rx_duration + in_study_tm +
## map_during.lme:     phtp_type + Rx_modal + Mob_level + Airway + opia + vaso +
## map_during.lme:     sed + relax + MAP_md_prior + MAP_CV_prior + (1 | record_id)
##                 npar    AIC    BIC  logLik deviance  Chisq Df Pr(>Chisq)
## map_during.lme0   25 3370.7 3477.8 -1660.3   3320.7
## map_during.lme    27 3374.6 3490.2 -1660.3   3320.6 0.1324  2      0.936
```

```
#
# MAP_after analysis
```

```r
#

vars_to_incl <- c("record_id", "MAP_md_after", "MAP_md_prior", "MAP_CV_prior",
                  "age",  "bmi", "Mob_level", "Rx_duration", "in_study_tm",
                  "Rx_sofa", "relax", "Rx_modal",
                  "vaso", "opia", "sed", "Airway", "phtp_type","sex")

map_after.lme <- lmer(MAP_md_after ~ age+sex+bmi+Rx_sofa+Rx_duration+
                        in_study_tm+phtp_type+Rx_modal+Mob_level+Airway+opia+
                        vaso+sed+relax+MAP_md_prior+MAP_CV_prior+(1|record_id),
                        data = na.omit(data0[ ,vars_to_incl]))

summary(map_after.lme, ddf="Kenward-Roger")
```

```
## Linear mixed model fit by REML. t-tests use Kenward-Roger's method [
## lmerModLmerTest]
## Formula:
## MAP_md_after ~ age + sex + bmi + Rx_sofa + Rx_duration + in_study_tm +
##     phtp_type + Rx_modal + Mob_level + Airway + opia + vaso +
##     sed + relax + MAP_md_prior + MAP_CV_prior + (1 | record_id)
##    Data: na.omit(data0[, vars_to_incl])
##
## REML criterion at convergence: 3528.2
##
## Scaled residuals:
##     Min      1Q  Median      3Q     Max
## -3.1792 -0.5887 -0.0461  0.5661  3.7094
##
## Random effects:
##  Groups    Name        Variance Std.Dev.
##  record_id (Intercept)  0.00    0.000
##  Residual              45.13    6.718
## Number of obs: 534, groups:  record_id, 107
##
## Fixed effects:
##                                                 Estimate Std. Error         df
## (Intercept)                                    16.181204   3.869732 151.712876
## age                                             0.000784   0.026035 158.270062
## sexfemale                                       0.498573   0.653766  37.620907
## bmi                                            -0.130905   0.066952 104.027908
## Rx_sofa                                        -0.199961   0.082039 104.602998
## Rx_duration                                    -0.011017   0.032681 505.400405
## in_study_tm                                     0.009813   0.028664  75.509822
## phtp_typecycling                               -0.366492   0.869685 340.166947
## phtp_typemobilisation                          -1.777871   3.098106 502.460844
## phtp_typerespiratory management                -0.692932   1.350984 475.062107
## phtp_typeexercise and respiratory management    0.323488   1.125795 479.667634
## phtp_typecomplex cycling and mobilisation       0.705011   2.473596 486.629837
## phtp_typecomplex exercise and mobilisation     -0.345341   2.988486 508.416210
## Rx_modalmixed                                   0.176243   1.633806 507.909637
## Rx_modalactive                                  1.436998   0.807620 361.214110
## Mob_leveledge-of-bed                           -1.349603   3.053623 505.773766
## Mob_levelout-of-bed                            -2.617592   3.229730 499.892314
## Airwaytracheostomy                              0.504575   1.154205 255.549824
```

```
## Airwaytube                                     1.163856   1.038509 477.487523
## opiayes                                       -0.064411   1.294030 344.537443
## vasoyes                                       -2.416665   0.719570 474.141353
## sedyes                                         1.025847   0.860583 396.124323
## relaxyes                                      -0.079076   0.844021 477.140501
## MAP_md_prior                                   0.849246   0.025969 223.143654
## MAP_CV_prior                                  -0.003919   0.010909 438.632097
##                                              t value Pr(>|t|)
## (Intercept)                                    4.181 4.88e-05 ***
## age                                            0.030 0.976014
## sexfemale                                      0.763 0.450447
## bmi                                           -1.955 0.053243 .
## Rx_sofa                                       -2.437 0.016480 *
## Rx_duration                                   -0.337 0.736185
## in_study_tm                                    0.342 0.733052
## phtp_typecycling                              -0.421 0.673723
## phtp_typemobilisation                         -0.574 0.566321
## phtp_typerespiratory management               -0.513 0.608253
## phtp_typeexercise and respiratory management   0.287 0.773975
## phtp_typecomplex cycling and mobilisation      0.285 0.775754
## phtp_typecomplex exercise and mobilisation    -0.116 0.908049
## Rx_modalmixed                                  0.108 0.914139
## Rx_modalactive                                 1.779 0.076032 .
## Mob_leveledge-of-bed                          -0.442 0.658702
## Mob_levelout-of-bed                           -0.810 0.418057
## Airwaytracheostomy                             0.437 0.662363
## Airwaytube                                     1.121 0.262979
## opiayes                                       -0.050 0.960330
## vasoyes                                       -3.358 0.000847 ***
## sedyes                                         1.192 0.233960
## relaxyes                                      -0.094 0.925395
## MAP_md_prior                                  32.702  < 2e-16 ***
## MAP_CV_prior                                  -0.359 0.719563
## ---
## Signif. codes:  0 '***' 0.001 '**' 0.01 '*' 0.05 '.' 0.1 ' ' 1
## convergence code: 0
## boundary (singular) fit: see ?isSingular
```

```
### Test for overall category
### phtp_type
map_after.lme0 <- lmer(MAP_md_after ~ age+sex+bmi+Rx_sofa+Rx_duration+
                       in_study_tm+Rx_modal+Mob_level+Airway+opia+
                       vaso+sed+relax+MAP_md_prior+MAP_CV_prior+(1|record_id),
                       data = na.omit(data0[ ,vars_to_incl]))

anova(map_after.lme, map_after.lme0, test="LRT")
```

```
## Data: na.omit(data0[, vars_to_incl])
## Models:
## map_after.lme0: MAP_md_after ~ age + sex + bmi + Rx_sofa + Rx_duration + in_study_tm +
## map_after.lme0:     Rx_modal + Mob_level + Airway + opia + vaso + sed + relax +
## map_after.lme0:     MAP_md_prior + MAP_CV_prior + (1 | record_id)
## map_after.lme: MAP_md_after ~ age + sex + bmi + Rx_sofa + Rx_duration + in_study_tm +
## map_after.lme:     phtp_type + Rx_modal + Mob_level + Airway + opia + vaso +
## map_after.lme:     sed + relax + MAP_md_prior + MAP_CV_prior + (1 | record_id)
```

```
##                 npar    AIC    BIC  logLik deviance  Chisq Df Pr(>Chisq)
## map_after.lme0   21 3568.1 3658.0 -1763.1   3526.1
## map_after.lme    27 3578.1 3693.6 -1762.0   3524.1 2.0405  6     0.9159
```

```
### Rx_modal
map_after.lme0 <- lmer(MAP_md_after ~ age+sex+bmi+Rx_sofa+Rx_duration+
                       in_study_tm+phtp_type+Mob_level+Airway+opia+
                       vaso+sed+relax+MAP_md_prior+MAP_CV_prior+(1|record_id),
                       data = na.omit(data0[ ,vars_to_incl]))

anova(map_after.lme, map_after.lme0, test="LRT")
```

```
## Data: na.omit(data0[, vars_to_incl])
## Models:
## map_after.lme0: MAP_md_after ~ age + sex + bmi + Rx_sofa + Rx_duration + in_study_tm +
## map_after.lme0:     phtp_type + Mob_level + Airway + opia + vaso + sed + relax +
## map_after.lme0:     MAP_md_prior + MAP_CV_prior + (1 | record_id)
## map_after.lme: MAP_md_after ~ age + sex + bmi + Rx_sofa + Rx_duration + in_study_tm +
## map_after.lme:     phtp_type + Rx_modal + Mob_level + Airway + opia + vaso +
## map_after.lme:     sed + relax + MAP_md_prior + MAP_CV_prior + (1 | record_id)
##                 npar    AIC    BIC  logLik deviance  Chisq Df Pr(>Chisq)
## map_after.lme0   25 3577.5 3684.5 -1763.8   3527.5
## map_after.lme    27 3578.1 3693.6 -1762.0   3524.1 3.4481  2     0.1783
```

```
### Airway
map_after.lme0 <- lmer(MAP_md_after ~ age+sex+bmi+Rx_sofa+Rx_duration+
                       in_study_tm+phtp_type+Rx_modal+Mob_level+opia+
                       vaso+sed+relax+MAP_md_prior+MAP_CV_prior+(1|record_id),
                       data = na.omit(data0[ ,vars_to_incl]))

anova(map_after.lme, map_after.lme0, test="LRT")
```

```
## Data: na.omit(data0[, vars_to_incl])
## Models:
## map_after.lme0: MAP_md_after ~ age + sex + bmi + Rx_sofa + Rx_duration + in_study_tm +
## map_after.lme0:     phtp_type + Rx_modal + Mob_level + opia + vaso + sed + relax +
## map_after.lme0:     MAP_md_prior + MAP_CV_prior + (1 | record_id)
## map_after.lme: MAP_md_after ~ age + sex + bmi + Rx_sofa + Rx_duration + in_study_tm +
## map_after.lme:     phtp_type + Rx_modal + Mob_level + Airway + opia + vaso +
## map_after.lme:     sed + relax + MAP_md_prior + MAP_CV_prior + (1 | record_id)
##                 npar    AIC    BIC  logLik deviance  Chisq Df Pr(>Chisq)
## map_after.lme0   25 3575.6 3682.6 -1762.8   3525.6
## map_after.lme    27 3578.1 3693.6 -1762.0   3524.1 1.5074  2     0.4706
```

```
### Mob_level
map_after.lme0 <- lmer(MAP_md_after ~ age+sex+bmi+Rx_sofa+Rx_duration+
                       in_study_tm+phtp_type+Rx_modal+Airway+opia+
                       vaso+sed+relax+MAP_md_prior+MAP_CV_prior+(1|record_id),
                       data = na.omit(data0[ ,vars_to_incl]))

anova(map_after.lme, map_after.lme0, test="LRT")
```

```
## Data: na.omit(data0[, vars_to_incl])
## Models:
## map_after.lme0: MAP_md_after ~ age + sex + bmi + Rx_sofa + Rx_duration + in_study_tm +
## map_after.lme0:     phtp_type + Rx_modal + Airway + opia + vaso + sed + relax +
```

```
## map_after.lme0:     MAP_md_prior + MAP_CV_prior + (1 | record_id)
## map_after.lme: MAP_md_after ~ age + sex + bmi + Rx_sofa + Rx_duration + in_study_tm +
## map_after.lme:     phtp_type + Rx_modal + Mob_level + Airway + opia + vaso +
## map_after.lme:     sed + relax + MAP_md_prior + MAP_CV_prior + (1 | record_id)
##                 npar    AIC    BIC  logLik deviance  Chisq Df Pr(>Chisq)
## map_after.lme0   25 3575.1 3682.2 -1762.6   3525.1
## map_after.lme    27 3578.1 3693.6 -1762.0   3524.1 1.0667  2     0.5866
```

```
#
# HR_during analysis
#

vars_to_incl <- c("record_id", "HR_md_during", "HR_md_prior", "HR_CV_prior",
                  "age",  "bmi", "Mob_level", "Rx_duration", "in_study_tm",
                  "Rx_sofa", "relax", "Rx_modal",
                  "vaso", "opia", "sed", "Airway", "phtp_type","sex")

hr_during.lme <- lmer(HR_md_during ~ age+sex+bmi+Rx_sofa+Rx_duration+
                       in_study_tm+phtp_type+Rx_modal+Mob_level+Airway+opia+
                       vaso+sed+relax+HR_md_prior+HR_CV_prior+(1|record_id),
                       data = na.omit(data0[ ,vars_to_incl]))

summary(hr_during.lme)
```

```
## Linear mixed model fit by REML. t-tests use Satterthwaite's method [
## lmerModLmerTest]
## Formula:
## HR_md_during ~ age + sex + bmi + Rx_sofa + Rx_duration + in_study_tm +
##     phtp_type + Rx_modal + Mob_level + Airway + opia + vaso +
##     sed + relax + HR_md_prior + HR_CV_prior + (1 | record_id)
##    Data: na.omit(data0[, vars_to_incl])
##
## REML criterion at convergence: 3321.6
##
## Scaled residuals:
##     Min      1Q  Median      3Q     Max
## -4.0956 -0.4635 -0.0425  0.4378  8.5006
##
## Random effects:
##  Groups    Name        Variance Std.Dev.
##  record_id (Intercept)  2.596   1.611
##  Residual              18.096   4.254
## Number of obs: 571, groups:  record_id, 107
##
## Fixed effects:
##                                               Estimate Std. Error         df
## (Intercept)                                  -0.549045   2.722041 201.480776
## age                                           0.018034   0.019941 108.092045
## sexfemale                                     0.241354   0.574611  74.865242
## bmi                                           0.016424   0.054084  84.224277
## Rx_sofa                                       0.002013   0.061962 188.925131
## Rx_duration                                   0.052758   0.020173 537.099916
## in_study_tm                                   0.024243   0.019897 401.588536
## phtp_typecycling                             -0.933612   0.593543 440.492029
## phtp_typemobilisation                         2.052609   2.002817 531.486451
```

```
## phtp_typerespiratory management                  0.717382   0.863392 542.792919
## phtp_typeexercise and respiratory management   0.288220   0.741981 545.998491
## phtp_typecomplex cycling and mobilisation     -2.587829   1.632340 541.131310
## phtp_typecomplex exercise and mobilisation     2.211190   1.943876 533.073702
## Rx_modalmixed                                 -0.380572   1.025824 542.511475
## Rx_modalactive                                 1.160637   0.538793 536.922332
## Mob_leveledge-of-bed                          -0.784166   1.977250 528.143543
## Mob_levelout-of-bed                            0.125171   2.076573 536.415788
## Airwaytracheostomy                            -0.897617   0.770438 488.649509
## Airwaytube                                    -0.488536   0.674013 544.781709
## opiayes                                        0.391255   0.755260 545.117417
## vasoyes                                       -0.111038   0.482169 522.195469
## sedyes                                         1.152326   0.554684 544.155570
## relaxyes                                      -0.192677   0.558089 545.988258
## HR_md_prior                                    0.964630   0.013950 373.624449
## HR_CV_prior                                    0.070475   0.020365 486.933075
##                                              t value Pr(>|t|)
## (Intercept)                                   -0.202 0.840352
## age                                            0.904 0.367813
## sexfemale                                      0.420 0.675667
## bmi                                            0.304 0.762130
## Rx_sofa                                        0.032 0.974115
## Rx_duration                                    2.615 0.009167 **
## in_study_tm                                    1.218 0.223774
## phtp_typecycling                              -1.573 0.116449
## phtp_typemobilisation                          1.025 0.305895
## phtp_typerespiratory management                0.831 0.406402
## phtp_typeexercise and respiratory management   0.388 0.697837
## phtp_typecomplex cycling and mobilisation     -1.585 0.113471
## phtp_typecomplex exercise and mobilisation     1.138 0.255834
## Rx_modalmixed                                 -0.371 0.710788
## Rx_modalactive                                 2.154 0.031673 *
## Mob_leveledge-of-bed                          -0.397 0.691827
## Mob_levelout-of-bed                            0.060 0.951957
## Airwaytracheostomy                            -1.165 0.244558
## Airwaytube                                    -0.725 0.468875
## opiayes                                        0.518 0.604641
## vasoyes                                       -0.230 0.817958
## sedyes                                         2.077 0.038229 *
## relaxyes                                      -0.345 0.730043
## HR_md_prior                                   69.150  < 2e-16 ***
## HR_CV_prior                                    3.461 0.000587 ***
## ---
## Signif. codes:  0 '***' 0.001 '**' 0.01 '*' 0.05 '.' 0.1 ' ' 1
```

```
### Test for overall category
### phtp_type
hr_during.lme0 <- lmer(HR_md_during ~ age+sex+bmi+Rx_sofa+Rx_duration+
                        in_study_tm+Rx_modal+Mob_level+Airway+opia+
                        vaso+sed+relax+HR_md_prior+HR_CV_prior+(1|record_id),
                        data = na.omit(data0[ ,vars_to_incl]))

anova(hr_during.lme, hr_during.lme0, test="LRT")
```

```
## Data: na.omit(data0[, vars_to_incl])
```

```
## Models:
## hr_during.lme0: HR_md_during ~ age + sex + bmi + Rx_sofa + Rx_duration + in_study_tm +
## hr_during.lme0:     Rx_modal + Mob_level + Airway + opia + vaso + sed + relax +
## hr_during.lme0:     HR_md_prior + HR_CV_prior + (1 | record_id)
## hr_during.lme: HR_md_during ~ age + sex + bmi + Rx_sofa + Rx_duration + in_study_tm +
## hr_during.lme:     phtp_type + Rx_modal + Mob_level + Airway + opia + vaso +
## hr_during.lme:     sed + relax + HR_md_prior + HR_CV_prior + (1 | record_id)
##                npar    AIC    BIC  logLik deviance  Chisq Df Pr(>Chisq)
## hr_during.lme0   21 3352.1 3443.4 -1655.1   3310.1
## hr_during.lme    27 3354.4 3471.8 -1650.2   3300.4 9.7393  6     0.1361
```

```
### Rx_modal
hr_during.lme0 <- lmer(HR_md_during ~ age+sex+bmi+Rx_sofa+Rx_duration+
                       in_study_tm+phtp_type+Mob_level+Airway+opia+
                       vaso+sed+relax+HR_md_prior+HR_CV_prior+(1|record_id),
                       data = na.omit(data0[ ,vars_to_incl]))

anova(hr_during.lme, hr_during.lme0, test="LRT")
```

```
## Data: na.omit(data0[, vars_to_incl])
## Models:
## hr_during.lme0: HR_md_during ~ age + sex + bmi + Rx_sofa + Rx_duration + in_study_tm +
## hr_during.lme0:     phtp_type + Mob_level + Airway + opia + vaso + sed + relax +
## hr_during.lme0:     HR_md_prior + HR_CV_prior + (1 | record_id)
## hr_during.lme: HR_md_during ~ age + sex + bmi + Rx_sofa + Rx_duration + in_study_tm +
## hr_during.lme:     phtp_type + Rx_modal + Mob_level + Airway + opia + vaso +
## hr_during.lme:     sed + relax + HR_md_prior + HR_CV_prior + (1 | record_id)
##                npar    AIC    BIC  logLik deviance  Chisq Df Pr(>Chisq)
## hr_during.lme0   25 3355.9 3464.6 -1652.9   3305.9
## hr_during.lme    27 3354.4 3471.8 -1650.2   3300.4 5.4983  2    0.06398 .
## ---
## Signif. codes:  0 '***' 0.001 '**' 0.01 '*' 0.05 '.' 0.1 ' ' 1
```

```
### Airway
hr_during.lme0 <- lmer(HR_md_during ~ age+sex+bmi+Rx_sofa+Rx_duration+
                       in_study_tm+phtp_type+Rx_modal+Mob_level+opia+
                       vaso+sed+relax+HR_md_prior+HR_CV_prior+(1|record_id),
                       data = na.omit(data0[ ,vars_to_incl]))

anova(hr_during.lme, hr_during.lme0, test="LRT")
```

```
## Data: na.omit(data0[, vars_to_incl])
## Models:
## hr_during.lme0: HR_md_during ~ age + sex + bmi + Rx_sofa + Rx_duration + in_study_tm +
## hr_during.lme0:     phtp_type + Rx_modal + Mob_level + opia + vaso + sed + relax +
## hr_during.lme0:     HR_md_prior + HR_CV_prior + (1 | record_id)
## hr_during.lme: HR_md_during ~ age + sex + bmi + Rx_sofa + Rx_duration + in_study_tm +
## hr_during.lme:     phtp_type + Rx_modal + Mob_level + Airway + opia + vaso +
## hr_during.lme:     sed + relax + HR_md_prior + HR_CV_prior + (1 | record_id)
##                npar    AIC    BIC  logLik deviance  Chisq Df Pr(>Chisq)
## hr_during.lme0   25 3351.8 3460.4 -1650.9   3301.8
## hr_during.lme    27 3354.4 3471.8 -1650.2   3300.4 1.3806  2     0.5014
```

```
### Mob_level
hr_during.lme0 <-lmer(HR_md_during ~ age+sex+bmi+Rx_sofa+Rx_duration+
                       in_study_tm+phtp_type+Rx_modal+Airway+opia+
```

```
                        vaso+sed+relax+HR_md_prior+HR_CV_prior+(1|record_id),
                        data = na.omit(data0[ ,vars_to_incl]))

anova(hr_during.lme, hr_during.lme0, test="LRT")
```

```
## Data: na.omit(data0[, vars_to_incl])
## Models:
## hr_during.lme0: HR_md_during ~ age + sex + bmi + Rx_sofa + Rx_duration + in_study_tm +
## hr_during.lme0:     phtp_type + Rx_modal + Airway + opia + vaso + sed + relax +
## hr_during.lme0:     HR_md_prior + HR_CV_prior + (1 | record_id)
## hr_during.lme: HR_md_during ~ age + sex + bmi + Rx_sofa + Rx_duration + in_study_tm +
## hr_during.lme:     phtp_type + Rx_modal + Mob_level + Airway + opia + vaso +
## hr_during.lme:     sed + relax + HR_md_prior + HR_CV_prior + (1 | record_id)
##                npar    AIC    BIC  logLik deviance  Chisq Df Pr(>Chisq)
## hr_during.lme0   25 3351.4 3460.1 -1650.7   3301.4
## hr_during.lme    27 3354.4 3471.8 -1650.2   3300.4 0.9897  2     0.6097
```

```
#
# HR_after analysis
#

vars_to_incl <- c("record_id", "HR_md_after", "HR_md_prior", "HR_CV_prior",
                  "age",  "bmi", "Mob_level", "Rx_duration", "in_study_tm",
                  "Rx_sofa", "relax", "Rx_modal",
                  "vaso", "opia", "sed", "Airway", "phtp_type","sex")

hr_after.lme <- lmer(HR_md_after ~ age+sex+bmi+Rx_sofa+Rx_duration+
                        in_study_tm+phtp_type+Rx_modal+Mob_level+Airway+opia+
                        vaso+sed+relax+HR_md_prior+HR_CV_prior+(1|record_id),
                        data = na.omit(data0[ ,vars_to_incl]))

summary(hr_after.lme)
```

```
## Linear mixed model fit by REML. t-tests use Satterthwaite's method [
## lmerModLmerTest]
## Formula: HR_md_after ~ age + sex + bmi + Rx_sofa + Rx_duration + in_study_tm +
##     phtp_type + Rx_modal + Mob_level + Airway + opia + vaso +
##     sed + relax + HR_md_prior + HR_CV_prior + (1 | record_id)
##    Data: na.omit(data0[, vars_to_incl])
##
## REML criterion at convergence: 3486.5
##
## Scaled residuals:
##     Min      1Q  Median      3Q     Max
## -4.4386 -0.5057 -0.0260  0.4408  8.5542
##
## Random effects:
##  Groups    Name        Variance Std.Dev.
##  record_id (Intercept)  0.5437  0.7373
##  Residual              26.7882  5.1757
## Number of obs: 569, groups:  record_id, 107
##
## Fixed effects:
##                                                Estimate Std. Error         df
```

```
## (Intercept)                                   0.321534   2.877470 138.362842
## age                                           0.047244   0.019927  77.701216
## sexfemale                                     0.474519   0.535703  30.764411
## bmi                                          -0.059489   0.052154  51.634867
## Rx_sofa                                      -0.131386   0.063282  68.507956
## Rx_duration                                   0.009084   0.023873 542.274152
## in_study_tm                                   0.034928   0.021340  79.375001
## phtp_typecycling                             -0.533260   0.663312 290.785753
## phtp_typemobilisation                         6.564446   2.373805 543.863181
## phtp_typerespiratory management               0.550887   1.001293 486.259150
## phtp_typeexercise and respiratory management  0.117915   0.866837 517.620185
## phtp_typecomplex cycling and mobilisation    -2.787128   1.916715 525.014328
## phtp_typecomplex exercise and mobilisation    4.525007   2.304624 543.691946
## Rx_modalmixed                                -0.945303   1.207917 539.585134
## Rx_modalactive                                0.968351   0.617537 365.263365
## Mob_leveledge-of-bed                         -5.849359   2.347490 543.845157
## Mob_levelout-of-bed                          -6.058677   2.450730 535.999663
## Airwaytracheostomy                            0.105170   0.863260 232.748264
## Airwaytube                                    0.866359   0.783676 490.614044
## opiayes                                       0.431557   0.875208 430.573364
## vasoyes                                       0.261051   0.552788 423.370056
## sedyes                                        1.323136   0.642686 406.216441
## relaxyes                                      0.184920   0.651759 514.202664
## HR_md_prior                                   0.962962   0.015199 164.980251
## HR_CV_prior                                   0.051771   0.023159 401.247826
##                                              t value Pr(>|t|)
## (Intercept)                                   0.112  0.91119
## age                                           2.371  0.02023 *
## sexfemale                                     0.886  0.38260
## bmi                                          -1.141  0.25928
## Rx_sofa                                      -2.076  0.04163 *
## Rx_duration                                   0.381  0.70372
## in_study_tm                                   1.637  0.10565
## phtp_typecycling                             -0.804  0.42209
## phtp_typemobilisation                         2.765  0.00588 **
## phtp_typerespiratory management               0.550  0.58245
## phtp_typeexercise and respiratory management  0.136  0.89185
## phtp_typecomplex cycling and mobilisation    -1.454  0.14651
## phtp_typecomplex exercise and mobilisation    1.963  0.05010 .
## Rx_modalmixed                                -0.783  0.43421
## Rx_modalactive                                1.568  0.11773
## Mob_leveledge-of-bed                         -2.492  0.01301 *
## Mob_levelout-of-bed                          -2.472  0.01374 *
## Airwaytracheostomy                            0.122  0.90314
## Airwaytube                                    1.106  0.26948
## opiayes                                       0.493  0.62220
## vasoyes                                       0.472  0.63700
## sedyes                                        2.059  0.04015 *
## relaxyes                                      0.284  0.77674
## HR_md_prior                                  63.357  < 2e-16 ***
## HR_CV_prior                                   2.235  0.02594 *
## ---
## Signif. codes:  0 '***' 0.001 '**' 0.01 '*' 0.05 '.' 0.1 ' ' 1
```

```r
### Test for overall category
### phtp_type
hr_after.lme0 <- lmer(HR_md_after ~ age+sex+bmi+Rx_sofa+Rx_duration+
                       in_study_tm+Rx_modal+Mob_level+Airway+opia+
                       vaso+sed+relax+HR_md_prior+HR_CV_prior+(1|record_id),
                       data = na.omit(data0[ ,vars_to_incl]))

anova(hr_after.lme, hr_after.lme0, test="LRT")
```

```
## Data: na.omit(data0[, vars_to_incl])
## Models:
## hr_after.lme0: HR_md_after ~ age + sex + bmi + Rx_sofa + Rx_duration + in_study_tm +
## hr_after.lme0:     Rx_modal + Mob_level + Airway + opia + vaso + sed + relax +
## hr_after.lme0:     HR_md_prior + HR_CV_prior + (1 | record_id)
## hr_after.lme: HR_md_after ~ age + sex + bmi + Rx_sofa + Rx_duration + in_study_tm +
## hr_after.lme:     phtp_type + Rx_modal + Mob_level + Airway + opia + vaso +
## hr_after.lme:     sed + relax + HR_md_prior + HR_CV_prior + (1 | record_id)
##               npar    AIC    BIC  logLik deviance  Chisq Df Pr(>Chisq)
## hr_after.lme0   21 3527.2 3618.4 -1742.6   3485.2
## hr_after.lme    27 3523.6 3640.8 -1734.8   3469.6 15.668  6    0.01565 *
## ---
## Signif. codes:  0 '***' 0.001 '**' 0.01 '*' 0.05 '.' 0.1 ' ' 1
```

```r
### Rx_modal
hr_after.lme0 <- lmer(HR_md_after ~ age+sex+bmi+Rx_sofa+Rx_duration+
                       in_study_tm+phtp_type+Mob_level+Airway+opia+
                       vaso+sed+relax+HR_md_prior+HR_CV_prior+(1|record_id),
                       data = na.omit(data0[ ,vars_to_incl]))

anova(hr_after.lme, hr_after.lme0, test="LRT")
```

```
## Data: na.omit(data0[, vars_to_incl])
## Models:
## hr_after.lme0: HR_md_after ~ age + sex + bmi + Rx_sofa + Rx_duration + in_study_tm +
## hr_after.lme0:     phtp_type + Mob_level + Airway + opia + vaso + sed + relax +
## hr_after.lme0:     HR_md_prior + HR_CV_prior + (1 | record_id)
## hr_after.lme: HR_md_after ~ age + sex + bmi + Rx_sofa + Rx_duration + in_study_tm +
## hr_after.lme:     phtp_type + Rx_modal + Mob_level + Airway + opia + vaso +
## hr_after.lme:     sed + relax + HR_md_prior + HR_CV_prior + (1 | record_id)
##               npar    AIC    BIC  logLik deviance Chisq Df Pr(>Chisq)
## hr_after.lme0   25 3523.1 3631.7 -1736.6   3473.1
## hr_after.lme    27 3523.6 3640.8 -1734.8   3469.6 3.584  2     0.1666
```

```r
### Airway
hr_after.lme0 <- lmer(HR_md_after ~ age+sex+bmi+Rx_sofa+Rx_duration+
                       in_study_tm+phtp_type+Rx_modal+Mob_level+opia+
                       vaso+sed+relax+HR_md_prior+HR_CV_prior+(1|record_id),
                       data = na.omit(data0[ ,vars_to_incl]))

anova(hr_after.lme, hr_after.lme0, test="LRT")
```

```
## Data: na.omit(data0[, vars_to_incl])
## Models:
## hr_after.lme0: HR_md_after ~ age + sex + bmi + Rx_sofa + Rx_duration + in_study_tm +
## hr_after.lme0:     phtp_type + Rx_modal + Mob_level + opia + vaso + sed + relax +
```

```
## hr_after.lme0:     HR_md_prior + HR_CV_prior + (1 | record_id)
## hr_after.lme: HR_md_after ~ age + sex + bmi + Rx_sofa + Rx_duration + in_study_tm +
## hr_after.lme:     phtp_type + Rx_modal + Mob_level + Airway + opia + vaso +
## hr_after.lme:     sed + relax + HR_md_prior + HR_CV_prior + (1 | record_id)
##                npar    AIC    BIC  logLik deviance  Chisq Df Pr(>Chisq)
## hr_after.lme0   25 3521.3 3629.9 -1735.7   3471.3
## hr_after.lme    27 3523.6 3640.8 -1734.8   3469.6 1.7534  2     0.4162
```

```r
### Mob_level
hr_after.lme0 <- lmer(HR_md_after ~ age+sex+bmi+Rx_sofa+Rx_duration+
                       in_study_tm+phtp_type+Rx_modal+Airway+opia+
                       vaso+sed+relax+HR_md_prior+HR_CV_prior+(1|record_id),
                       data = na.omit(data0[ ,vars_to_incl]))

anova(hr_after.lme, hr_after.lme0, test="LRT")
```

```
## Data: na.omit(data0[, vars_to_incl])
## Models:
## hr_after.lme0: HR_md_after ~ age + sex + bmi + Rx_sofa + Rx_duration + in_study_tm +
## hr_after.lme0:     phtp_type + Rx_modal + Airway + opia + vaso + sed + relax +
## hr_after.lme0:     HR_md_prior + HR_CV_prior + (1 | record_id)
## hr_after.lme: HR_md_after ~ age + sex + bmi + Rx_sofa + Rx_duration + in_study_tm +
## hr_after.lme:     phtp_type + Rx_modal + Mob_level + Airway + opia + vaso +
## hr_after.lme:     sed + relax + HR_md_prior + HR_CV_prior + (1 | record_id)
##                npar    AIC    BIC  logLik deviance  Chisq Df Pr(>Chisq)
## hr_after.lme0   25 3526.8 3635.4 -1738.4   3476.8
## hr_after.lme    27 3523.6 3640.8 -1734.8   3469.6 7.2129  2    0.02715 *
## ---
## Signif. codes:  0 '***' 0.001 '**' 0.01 '*' 0.05 '.' 0.1 ' ' 1
```

```r
#
# SpO2_during analysis
#

vars_to_incl <- c("record_id", "SpO2_md_during", "SpO2_md_prior",
                  "SpO2_CV_prior","age",  "bmi","Mob_level", "Rx_duration",
                  "in_study_tm", "Rx_sofa", "relax", "Rx_modal",
                  "vaso", "opia", "sed", "Airway", "phtp_type","sex")


spo2_during.lme <- lmer(SpO2_md_during ~ age+sex+bmi+Rx_sofa+Rx_duration+
                       in_study_tm+phtp_type+Rx_modal+Mob_level+Airway+opia+
                       vaso+sed+relax+SpO2_md_prior+SpO2_CV_prior+(1|record_id),
                       data = na.omit(data0[ ,vars_to_incl]))

summary(spo2_during.lme)
```

```
## Linear mixed model fit by REML. t-tests use Satterthwaite's method [
## lmerModLmerTest]
## Formula:
## SpO2_md_during ~ age + sex + bmi + Rx_sofa + Rx_duration + in_study_tm +
##     phtp_type + Rx_modal + Mob_level + Airway + opia + vaso +
##     sed + relax + SpO2_md_prior + SpO2_CV_prior + (1 | record_id)
##    Data: na.omit(data0[, vars_to_incl])
##
## REML criterion at convergence: 1923.2
```

```
##
## Scaled residuals:
##     Min      1Q  Median      3Q     Max
## -5.0245 -0.5701  0.0708  0.5743  3.1244
##
## Random effects:
##  Groups    Name        Variance Std.Dev.
##  record_id (Intercept) 0.008125 0.09014
##  Residual              1.539372 1.24071
## Number of obs: 570, groups:  record_id, 107
##
## Fixed effects:
##                                                Estimate Std. Error         df
## (Intercept)                                   19.369938   2.504350 453.057073
## age                                            0.001475   0.004529 118.317143
## sexfemale                                     -0.081794   0.116965  36.651929
## bmi                                            0.005622   0.012042  84.698856
## Rx_sofa                                       -0.008635   0.014599  93.172226
## Rx_duration                                   -0.009522   0.005710 540.625229
## in_study_tm                                    0.008723   0.004963  97.116819
## phtp_typecycling                               0.123902   0.156777 343.100495
## phtp_typemobilisation                          0.217871   0.564795 541.746955
## phtp_typerespiratory management                0.045549   0.238728 499.057901
## phtp_typeexercise and respiratory management   0.524199   0.205898 514.826930
## phtp_typecomplex cycling and mobilisation      0.214398   0.455483 527.284085
## phtp_typecomplex exercise and mobilisation     0.325765   0.548072 544.598711
## Rx_modalmixed                                  0.297288   0.287192 538.596512
## Rx_modalactive                                 0.014216   0.145584 368.708666
## Mob_leveledge-of-bed                          -0.639079   0.559617 543.723130
## Mob_levelout-of-bed                           -1.027433   0.583006 531.457180
## Airwaytracheostomy                             0.393988   0.200626 239.308875
## Airwaytube                                     0.047035   0.184038 489.159167
## opiayes                                       -0.085673   0.207137 428.966229
## vasoyes                                       -0.136168   0.129465 461.429682
## sedyes                                         0.080751   0.148392 378.409045
## relaxyes                                       0.138123   0.153839 513.583067
## SpO2_md_prior                                  0.796806   0.024619 472.553999
## SpO2_CV_prior                                  0.009217   0.018494 544.730071
##                                              t value Pr(>|t|)
## (Intercept)                                    7.735 6.79e-14 ***
## age                                            0.326   0.7452
## sexfemale                                     -0.699   0.4888
## bmi                                            0.467   0.6418
## Rx_sofa                                       -0.592   0.5556
## Rx_duration                                   -1.667   0.0960 .
## in_study_tm                                    1.758   0.0820 .
## phtp_typecycling                               0.790   0.4299
## phtp_typemobilisation                          0.386   0.6998
## phtp_typerespiratory management                0.191   0.8488
## phtp_typeexercise and respiratory management   2.546   0.0112 *
## phtp_typecomplex cycling and mobilisation      0.471   0.6380
## phtp_typecomplex exercise and mobilisation     0.594   0.5525
## Rx_modalmixed                                  1.035   0.3011
## Rx_modalactive                                 0.098   0.9223
```

```
## Mob_leveledge-of-bed                              -1.142   0.2540
## Mob_levelout-of-bed                               -1.762   0.0786 .
## Airwaytracheostomy                                 1.964   0.0507 .
## Airwaytube                                         0.256   0.7984
## opiayes                                           -0.414   0.6794
## vasoyes                                           -1.052   0.2935
## sedyes                                             0.544   0.5866
## relaxyes                                           0.898   0.3697
## SpO2_md_prior                                     32.366  < 2e-16 ***
## SpO2_CV_prior                                      0.498   0.6184
## ---
## Signif. codes:  0 '***' 0.001 '**' 0.01 '*' 0.05 '.' 0.1 ' ' 1
```

```
### Test for overall category
### phtp_type
spo2_during.lme0 <- lmer(SpO2_md_during ~ age+sex+bmi+Rx_sofa+Rx_duration+
                       in_study_tm+phtp_type+Rx_modal+Mob_level+Airway+opia+
                       vaso+sed+relax+SpO2_md_prior+SpO2_CV_prior+(1|record_id),
                       data = na.omit(data0[ ,vars_to_incl]))

anova(spo2_during.lme, spo2_during.lme0, test="LRT")
```

```
## Data: na.omit(data0[, vars_to_incl])
## Models:
## spo2_during.lme: SpO2_md_during ~ age + sex + bmi + Rx_sofa + Rx_duration + in_study_tm +
## spo2_during.lme:     phtp_type + Rx_modal + Mob_level + Airway + opia + vaso +
## spo2_during.lme:     sed + relax + SpO2_md_prior + SpO2_CV_prior + (1 | record_id)
## spo2_during.lme0: SpO2_md_during ~ age + sex + bmi + Rx_sofa + Rx_duration + in_study_tm +
## spo2_during.lme0:     phtp_type + Rx_modal + Mob_level + Airway + opia + vaso +
## spo2_during.lme0:     sed + relax + SpO2_md_prior + SpO2_CV_prior + (1 | record_id)
##                  npar    AIC    BIC  logLik deviance Chisq Df Pr(>Chisq)
## spo2_during.lme    27 1894.5 2011.8 -920.25   1840.5
## spo2_during.lme0   27 1894.5 2011.8 -920.25   1840.5     0  0          1
```

```
### Rx_modal
spo2_during.lme0 <- lmer(SpO2_md_during ~ age+sex+bmi+Rx_sofa+Rx_duration+
                       in_study_tm+Rx_modal+Mob_level+Airway+opia+
                       vaso+sed+relax+SpO2_md_prior+SpO2_CV_prior+(1|record_id),
                       data = na.omit(data0[ ,vars_to_incl]))

anova(spo2_during.lme, spo2_during.lme0, test="LRT")
```

```
## Data: na.omit(data0[, vars_to_incl])
## Models:
## spo2_during.lme0: SpO2_md_during ~ age + sex + bmi + Rx_sofa + Rx_duration + in_study_tm +
## spo2_during.lme0:     Rx_modal + Mob_level + Airway + opia + vaso + sed + relax +
## spo2_during.lme0:     SpO2_md_prior + SpO2_CV_prior + (1 | record_id)
## spo2_during.lme: SpO2_md_during ~ age + sex + bmi + Rx_sofa + Rx_duration + in_study_tm +
## spo2_during.lme:     phtp_type + Rx_modal + Mob_level + Airway + opia + vaso +
## spo2_during.lme:     sed + relax + SpO2_md_prior + SpO2_CV_prior + (1 | record_id)
##                  npar    AIC    BIC  logLik deviance  Chisq Df Pr(>Chisq)
## spo2_during.lme0   21 1889.8 1981.0 -923.89   1847.8
## spo2_during.lme    27 1894.5 2011.8 -920.25   1840.5 7.2697  6     0.2966
```

```
### Airway
spo2_during.lme <- lmer(SpO2_md_during ~ age+sex+bmi+Rx_sofa+Rx_duration+
```

```r
                        in_study_tm+phtp_type+Rx_modal+Mob_level+opia+
                        vaso+sed+relax+SpO2_md_prior+SpO2_CV_prior+(1|record_id),
                        data = na.omit(data0[ ,vars_to_incl]))

anova(spo2_during.lme, spo2_during.lme0, test="LRT")
```

```
## Data: na.omit(data0[, vars_to_incl])
## Models:
## spo2_during.lme0: SpO2_md_during ~ age + sex + bmi + Rx_sofa + Rx_duration + in_study_tm +
## spo2_during.lme0:     Rx_modal + Mob_level + Airway + opia + vaso + sed + relax +
## spo2_during.lme0:     SpO2_md_prior + SpO2_CV_prior + (1 | record_id)
## spo2_during.lme: SpO2_md_during ~ age + sex + bmi + Rx_sofa + Rx_duration + in_study_tm +
## spo2_during.lme:     phtp_type + Rx_modal + Mob_level + opia + vaso + sed + relax +
## spo2_during.lme:     SpO2_md_prior + SpO2_CV_prior + (1 | record_id)
##                  npar    AIC    BIC  logLik deviance  Chisq Df Pr(>Chisq)
## spo2_during.lme0   21 1889.8 1981.0 -923.89   1847.8
## spo2_during.lme    25 1896.5 2005.2 -923.27   1846.5 1.2334  4     0.8726
```

```r
### Mob_level
spo2_during.lme0 <- lmer(SpO2_md_during ~ age+sex+bmi+Rx_sofa+Rx_duration+
                        in_study_tm+phtp_type+Rx_modal+Airway+opia+
                        vaso+sed+relax+SpO2_md_prior+SpO2_CV_prior+(1|record_id),
                        data = na.omit(data0[ ,vars_to_incl]))


anova(spo2_during.lme, spo2_during.lme0, test="LRT")
```

```
## Data: na.omit(data0[, vars_to_incl])
## Models:
## spo2_during.lme: SpO2_md_during ~ age + sex + bmi + Rx_sofa + Rx_duration + in_study_tm +
## spo2_during.lme:     phtp_type + Rx_modal + Mob_level + opia + vaso + sed + relax +
## spo2_during.lme:     SpO2_md_prior + SpO2_CV_prior + (1 | record_id)
## spo2_during.lme0: SpO2_md_during ~ age + sex + bmi + Rx_sofa + Rx_duration + in_study_tm +
## spo2_during.lme0:     phtp_type + Rx_modal + Airway + opia + vaso + sed + relax +
## spo2_during.lme0:     SpO2_md_prior + SpO2_CV_prior + (1 | record_id)
##                  npar    AIC    BIC  logLik deviance  Chisq Df Pr(>Chisq)
## spo2_during.lme    25 1896.5 2005.2 -923.27   1846.5
## spo2_during.lme0   25 1894.8 2003.5 -922.42   1844.8 1.6981  0  < 2.2e-16 ***
## ---
## Signif. codes:  0 '***' 0.001 '**' 0.01 '*' 0.05 '.' 0.1 ' ' 1
```

```r
#
# SpO2_after analysis
#

vars_to_incl <- c("record_id", "SpO2_md_after", "SpO2_md_prior",
                  "SpO2_CV_prior","age",  "bmi", "Mob_level", "Rx_duration",
                  "in_study_tm", "Rx_sofa", "relax", "Rx_modal",
                  "vaso", "opia", "sed", "Airway", "phtp_type","sex")

spo2_after.lme <- lmer(SpO2_md_after ~ age+sex+bmi+Rx_sofa+Rx_duration+
                        in_study_tm+phtp_type+Rx_modal+Mob_level+Airway+opia+
                        vaso+sed+relax+SpO2_md_prior+SpO2_CV_prior+(1|record_id),
                        data = na.omit(data0[ ,vars_to_incl]))
```

```r
summary(spo2_after.lme)
```

```
## Linear mixed model fit by REML. t-tests use Satterthwaite's method [
## lmerModLmerTest]
## Formula:
## SpO2_md_after ~ age + sex + bmi + Rx_sofa + Rx_duration + in_study_tm +
##     phtp_type + Rx_modal + Mob_level + Airway + opia + vaso +
##     sed + relax + SpO2_md_prior + SpO2_CV_prior + (1 | record_id)
##    Data: na.omit(data0[, vars_to_incl])
##
## REML criterion at convergence: 2156.4
##
## Scaled residuals:
##     Min      1Q  Median      3Q     Max
## -4.0597 -0.5798  0.0913  0.5933  3.8531
##
## Random effects:
##  Groups    Name        Variance Std.Dev.
##  record_id (Intercept) 0.01278  0.1131
##  Residual              2.39411  1.5473
## Number of obs: 568, groups:  record_id, 107
##
## Fixed effects:
##                                              Estimate Std. Error         df
## (Intercept)                                 26.099836   3.129390 448.473549
## age                                          0.004203   0.005710 117.142805
## sexfemale                                   -0.145858   0.146365  34.393448
## bmi                                          0.005944   0.015071  80.705111
## Rx_sofa                                     -0.007487   0.018217  87.918005
## Rx_duration                                  0.008297   0.007131 538.236838
## in_study_tm                                  0.011347   0.006208  94.423327
## phtp_typecycling                            -0.073259   0.195568 334.591707
## phtp_typemobilisation                       -0.816121   0.704694 539.663248
## phtp_typerespiratory management             -0.429787   0.297737 494.315530
## phtp_typeexercise and respiratory management  0.351105   0.258572 513.144086
## phtp_typecomplex cycling and mobilisation   -0.300738   0.568077 524.397476
## phtp_typecomplex exercise and mobilisation  -0.974758   0.683599 542.571542
## Rx_modalmixed                                0.127408   0.358231 536.297176
## Rx_modalactive                              -0.047003   0.181903 359.308889
## Mob_leveledge-of-bed                         0.557083   0.698086 541.680474
## Mob_levelout-of-bed                          0.131273   0.727238 528.521132
## Airwaytracheostomy                           0.047379   0.250647 229.129475
## Airwaytube                                  -0.439886   0.229646 483.142871
## opiayes                                     -0.127616   0.258406 423.529408
## vasoyes                                     -0.181384   0.161703 455.821998
## sedyes                                      -0.072951   0.185990 377.856515
## relaxyes                                    -0.072394   0.191868 509.666598
## SpO2_md_prior                                0.728914   0.030769 468.490012
## SpO2_CV_prior                               -0.003280   0.023133 542.603605
##                                             t value Pr(>|t|)
## (Intercept)                                   8.340  9.2e-16 ***
## age                                           0.736   0.4631
## sexfemale                                    -0.997   0.3259
## bmi                                           0.394   0.6943
```

```
## Rx_sofa                                       -0.411   0.6821
## Rx_duration                                    1.164   0.2451
## in_study_tm                                    1.828   0.0707 .
## phtp_typecycling                              -0.375   0.7082
## phtp_typemobilisation                         -1.158   0.2473
## phtp_typerespiratory management               -1.444   0.1495
## phtp_typeexercise and respiratory management   1.358   0.1751
## phtp_typecomplex cycling and mobilisation     -0.529   0.5968
## phtp_typecomplex exercise and mobilisation    -1.426   0.1545
## Rx_modalmixed                                  0.356   0.7222
## Rx_modalactive                                -0.258   0.7963
## Mob_leveledge-of-bed                           0.798   0.4252
## Mob_levelout-of-bed                            0.181   0.8568
## Airwaytracheostomy                             0.189   0.8502
## Airwaytube                                    -1.915   0.0560 .
## opiayes                                       -0.494   0.6217
## vasoyes                                       -1.122   0.2626
## sedyes                                        -0.392   0.6951
## relaxyes                                      -0.377   0.7061
## SpO2_md_prior                                 23.690  < 2e-16 ***
## SpO2_CV_prior                                 -0.142   0.8873
## ---
## Signif. codes:  0 '***' 0.001 '**' 0.01 '*' 0.05 '.' 0.1 ' ' 1
```

```r
### Test for overall category
### phtp_type
spo2_after.lme0 <- lmer(SpO2_md_after ~ age+sex+bmi+Rx_sofa+Rx_duration+
                       in_study_tm+Rx_modal+Mob_level+Airway+opia+
                       vaso+sed+relax+SpO2_md_prior+SpO2_CV_prior+(1|record_id),
                       data = na.omit(data0[ ,vars_to_incl]))

anova(spo2_after.lme, spo2_after.lme0, test="LRT")
```

```
## Data: na.omit(data0[, vars_to_incl])
## Models:
## spo2_after.lme0: SpO2_md_after ~ age + sex + bmi + Rx_sofa + Rx_duration + in_study_tm +
## spo2_after.lme0:     Rx_modal + Mob_level + Airway + opia + vaso + sed + relax +
## spo2_after.lme0:     SpO2_md_prior + SpO2_CV_prior + (1 | record_id)
## spo2_after.lme: SpO2_md_after ~ age + sex + bmi + Rx_sofa + Rx_duration + in_study_tm +
## spo2_after.lme:     phtp_type + Rx_modal + Mob_level + Airway + opia + vaso +
## spo2_after.lme:     sed + relax + SpO2_md_prior + SpO2_CV_prior + (1 | record_id)
##                 npar    AIC    BIC  logLik deviance  Chisq Df Pr(>Chisq)
## spo2_after.lme0   21 2134.4 2225.6 -1046.2   2092.4
## spo2_after.lme    27 2138.8 2256.1 -1042.4   2084.8 7.5438  6     0.2735
```

```r
### Rx_modal
spo2_after.lme0 <- lmer(SpO2_md_after ~ age+sex+bmi+Rx_sofa+Rx_duration+
                       in_study_tm+phtp_type+Mob_level+Airway+opia+
                       vaso+sed+relax+SpO2_md_prior+SpO2_CV_prior+(1|record_id),
                       data = na.omit(data0[ ,vars_to_incl]))

anova(spo2_after.lme, spo2_after.lme0, test="LRT")
```

```
## Data: na.omit(data0[, vars_to_incl])
## Models:
```

```
## spo2_after.lme0: SpO2_md_after ~ age + sex + bmi + Rx_sofa + Rx_duration + in_study_tm +
## spo2_after.lme0:     phtp_type + Mob_level + Airway + opia + vaso + sed + relax +
## spo2_after.lme0:     SpO2_md_prior + SpO2_CV_prior + (1 | record_id)
## spo2_after.lme: SpO2_md_after ~ age + sex + bmi + Rx_sofa + Rx_duration + in_study_tm +
## spo2_after.lme:     phtp_type + Rx_modal + Mob_level + Airway + opia + vaso +
## spo2_after.lme:     sed + relax + SpO2_md_prior + SpO2_CV_prior + (1 | record_id)
##                 npar    AIC    BIC  logLik deviance  Chisq Df Pr(>Chisq)
## spo2_after.lme0   25 2135.1 2243.6 -1042.5   2085.1
## spo2_after.lme    27 2138.8 2256.1 -1042.4   2084.8 0.2381  2     0.8878
```

```
### Airway
spo2_after.lme0 <- lmer(SpO2_md_after ~ age+sex+bmi+Rx_sofa+Rx_duration+
                      in_study_tm+phtp_type+Rx_modal+Mob_level+opia+
                      vaso+sed+relax+SpO2_md_prior+SpO2_CV_prior+(1|record_id),
                      data = na.omit(data0[ ,vars_to_incl]))

anova(spo2_after.lme, spo2_after.lme0, test="LRT")
```

```
## Data: na.omit(data0[, vars_to_incl])
## Models:
## spo2_after.lme0: SpO2_md_after ~ age + sex + bmi + Rx_sofa + Rx_duration + in_study_tm +
## spo2_after.lme0:     phtp_type + Rx_modal + Mob_level + opia + vaso + sed + relax +
## spo2_after.lme0:     SpO2_md_prior + SpO2_CV_prior + (1 | record_id)
## spo2_after.lme: SpO2_md_after ~ age + sex + bmi + Rx_sofa + Rx_duration + in_study_tm +
## spo2_after.lme:     phtp_type + Rx_modal + Mob_level + Airway + opia + vaso +
## spo2_after.lme:     sed + relax + SpO2_md_prior + SpO2_CV_prior + (1 | record_id)
##                 npar    AIC    BIC  logLik deviance  Chisq Df Pr(>Chisq)
## spo2_after.lme0   25 2142.6 2251.1 -1046.3   2092.6
## spo2_after.lme    27 2138.8 2256.1 -1042.4   2084.8 7.7256  2    0.02101 *
## ---
## Signif. codes:  0 '***' 0.001 '**' 0.01 '*' 0.05 '.' 0.1 ' ' 1
```

```
### Mob_level
spo2_after.lme0 <- lmer(SpO2_md_after ~ age+sex+bmi+Rx_sofa+Rx_duration+
                      in_study_tm+phtp_type+Rx_modal+Airway+opia+
                      vaso+sed+relax+SpO2_md_prior+SpO2_CV_prior+(1|record_id),
                      data = na.omit(data0[ ,vars_to_incl]))

anova(spo2_after.lme, spo2_after.lme0, test="LRT")
```

```
## Data: na.omit(data0[, vars_to_incl])
## Models:
## spo2_after.lme0: SpO2_md_after ~ age + sex + bmi + Rx_sofa + Rx_duration + in_study_tm +
## spo2_after.lme0:     phtp_type + Rx_modal + Airway + opia + vaso + sed + relax +
## spo2_after.lme0:     SpO2_md_prior + SpO2_CV_prior + (1 | record_id)
## spo2_after.lme: SpO2_md_after ~ age + sex + bmi + Rx_sofa + Rx_duration + in_study_tm +
## spo2_after.lme:     phtp_type + Rx_modal + Mob_level + Airway + opia + vaso +
## spo2_after.lme:     sed + relax + SpO2_md_prior + SpO2_CV_prior + (1 | record_id)
##                 npar    AIC    BIC  logLik deviance  Chisq Df Pr(>Chisq)
## spo2_after.lme0   25 2137.1 2245.6 -1043.5   2087.1
## spo2_after.lme    27 2138.8 2256.1 -1042.4   2084.8 2.2179  2     0.3299
```
