# Supplementary figures and images for: Cardiorespiratory response to early rehabilitation in critically ill adults: A secondary analysis of a randomised controlled trial
Source: PLoS One. 2022 Feb 3;17(2):e0262779. doi: 10.1371/journal.pone.0262779 (PMC8812982; doi:10.1371/journal.pone.0262779)

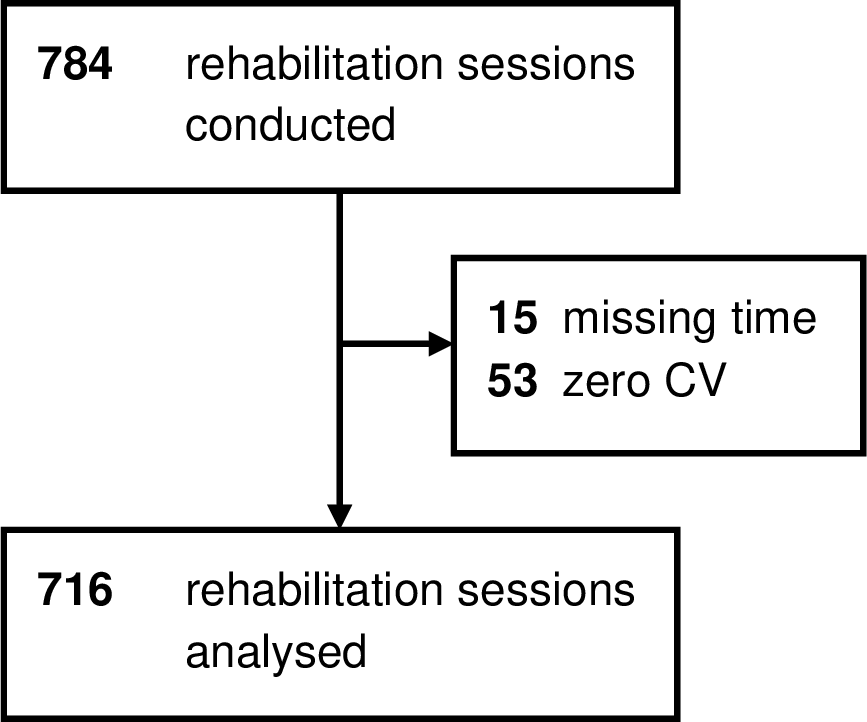

Supplement: S1 Fig — (TIF) [file pone.0262779.s008.tif]
