# Supplementary material for: Cardiorespiratory response to early rehabilitation in critically ill adults: A secondary analysis of a randomised controlled trial
Source: PLoS One. 2022 Feb 3;17(2):e0262779. doi: 10.1371/journal.pone.0262779 (PMC8812982; doi:10.1371/journal.pone.0262779)
Supplement: Attachment — Submitted filename: Response to Reviewers.pdf [file pone.0262779.s010.pdf]

Responses to Reviewers

The reviewers' comments are shown in **bold** type, our responses in plain type and changed passages of the manuscript are shown in *cursive* type. All pages and lines are in reference to the revised manuscript in the provided PDF-proof.

Reviewer #2:

Thank you for your comprehensive response and changes to the manuscript.
I think it would be good to clarify that "clinically relevant" (if I understand correctly) is related to safety - not sure this is entirely clear.
Perhaps line 431 should read "negatively effect physiological parameters" linking it back to safety. I think this is important as the next step would then be navigating the line between safety and efficacy (intensity)

We appreciate the reviewer's comment and changed the manuscript as follows:

Lines 211-213:
"The safety cut-off for a clinically relevant variation in physiological measurements was defined as >10% variation – based on half of what is commonly reported as an adverse event (>20%) in the literature [22]."

We would further like to mention, that above sentence is written under the subheading 'safety analysis' which should make it very clear to readers that this is about safety.

Lines 421-422:
"Finally, the safety cut-off for a clinically relevant variation was chosen in absence of previous evidence."

Lines 431-432:
"[…], our findings indicate that rehabilitation in the ICU is safe and does not negatively influence physiological parameters."

We would further like to highlight the following (unchanged) passage within our manuscript which elucidates that 'clinically relevant variations' are in relation to safety:

Lines 109-112:
"Finally, we re-evaluated safety by examining sessions with clinically relevant variations (>10%), an adverse event or therapy discontinuation to determine predictors and explored individual characteristics of patients with a strong physiological reaction."

We thank the reviewer for their time and dedication to improve our manuscript. We hope to have fully satisfied all concerns in order to see this manuscript published.
